# Unravelling the moons: review of the genera *Paratetilla* and *Cinachyrella* in the Indo-Pacific (Demospongiae, Tetractinellida, Tetillidae)

**DOI:** 10.3897/zookeys.791.27546

**Published:** 2018-10-22

**Authors:** Nadiezhda Santodomingo, Leontine E. Becking

**Affiliations:** 1 Department of Life Sciences, The Natural History Museum, Cromwell Road, SW7 5BD London, UK The Natural History Museum London United Kingdom; 2 Naturalis Biodiversity Center, P.O. Box 9517, 2300 RA Leiden, The Netherlands Wageningen University Wageningen Netherlands; 3 Marine Animal Ecology, Wageningen University, De Elst 1, 6708 WD, Wageningen, The Netherlands Naturalis Biodiversity Center Leiden Netherlands; 4 Wageningen Marine Research, Ankerpark 27, Den Helder, The Netherlands Wageningen Marine Research Den Helder Netherlands

**Keywords:** anchialine systems, coral reef, mangrove, marine lake, Porifera

## Abstract

*Paratetillabacca* (Selenka, 1867) and *Cinachyrellaaustraliensis* (Carter, 1886) occur in a broad range of marine environments and are allegedly widely distributed species in the Indo-Pacific. We coin the term ‘moon sponges’ for these species as they are spherical in shape with numerous porocalices resembling the lunar surface. Both species have a complex taxonomic history with high synonymization, in particular by Burton (1934, 1959). An examination of the junior synonyms proposed by Burton (1934, 1959) was conducted to establish the validity of the names. More than 230 specimens from Naturalis Biodiversity Center were reviewed that belong to the genera *Paratetilla* and *Cinachyrella* from marine lakes, coral reefs, and mangroves in Indonesia. The aim of the current study was to untangle the taxonomic history, describe the collection of moon sponges from Indonesia, and develop a key. We extensively reviewed the taxonomic literature as well as holotypes of most of the species synonymized by Burton. The taxonomic history of *Paratetilla* spp. and *Cinachyrellaaustraliensis* showed some cases of misinterpreted synonyms, misidentifications, and lack of detailed descriptions for some species. The conclusion of the revision is that there are three valid species of *Paratetilla* (*P.arcifera*, *P.bacca*, and *P.corrugata*) and four valid species of *Cinachyrella* (*C.australiensis*, *C.porosa*, *C.paterifera*, and *C.schulzei*) in Indonesia. This is furthermore corroborated by molecular work from previous studies. *Paratetillaarcifera*Wilson 1925 and *C.porosa* (Lendenfeld, 1888) are resurrected. A full review of taxonomic history is provided as well as a key for identification of moon sponges from Indonesia. All species are sympatric and we expect that there are undescribed species remaining within the Tetillidae from the Indo-Pacific. Our current review provides the framework from which to describe new species in the genera *Paratetilla* and *Cinachyrella* from the Indo-Pacific.

## Introduction

Moon sponges include two good examples of allegedly widely distributed species in the Indo-Pacific: *Paratetillabacca* (Selenka, 1867) and *Cinachyrellaaustraliensis* (Carter, 1886). They are conspicuous dwellers of a broad range of marine environments, including coral reefs, rocky shores, and coastal mangroves, as well as landlocked marine systems called marine lakes (e.g. Hooper et al. 2000, de Voogd and Cleary 2008, de Voogd et al. 2009, Becking et al. 2011). We use the term ‘moon sponges’ as these species are spherical in shape with numerous porocalices resembling the lunar surface and colored various shades of yellow, orange and brown. This common name has now been adopted by different authors (e.g., Szitenberg et al. 2013). Naturalis Biodiversity Center houses hundreds of moon sponges with a great diversity in morphology that were collected in Indonesia from 2006–2011 with the aim to survey the sponge biodiversity.

The genera *Paratetilla* and *Cinachyrella*, belong to the family Tetillidae, suborder Spirophorina, order Tetractinellida, class Demospongiae. As spirophorids, they are characterized by the presence of rugose sigmaspires (van Soest and Hooper 2002). Similar to most tetillids, their globular shape is composed of triaenes and oxeas arranged in a radiate skeleton. Recent revisions of the order and the family have been compiled in the Systema Porifera by van Soest and Hooper (2002) and van Soest and Rützler (2002), respectively. Although 26 nominal genera have been described, only ten valid genera are recognized, which are differentiated by the presence of cortical structures, specialized pore-sieves (porocalices) and composition of the complementary spicules (Rützler 1987, van Soest and Rützler 2002, Carella et al. 2016) (Table 1). The principal types of spicules of this family are: 1. *Megascleres*, oxeas and triaenes (pro-, plagio, ortho, and anatriaenes), and 2. *Microscleres*, microxeas and sigmaspires. Identification at species level is mainly based on the geometry and size range of all spicule types and presence/absence of triaenes (van Soest 1977, Rützler 1987, Rützler and Smith 1992, Lazoski et al. 1999, de Voogd and van Soest 2007, Carella et al. 2016).

**Table 1.** Valid genera of Tetillidae Sollas, 1888 and principal characteristics used to distinguish them. (+) present, (-) absent. (AN) Antarctic, (AT) Atlantic, (CA) Caribbean, (IP) Indo-Pacific. Modified from Rützler (1987), van Soest and Rützler (2002), Carella et al. (2016). Number of valid species consulted at the World Porifera Database (van Soest et al. 2018; accessed 04 Jun 2018).

Genus	Cortex (reinforced by)	Porocalices (shape)	Accessory spicules	Valid species	Distribution
*Tetilla* Schmidt, 1868	–	–	–	54	AT, CA, IP
*Craniella* Schmidt, 1870	+ (minute smooth oxea)	–	–	42	AN, AT, CA, IP
*Cinachyra* Sollas, 1886	+ (minute smooth oxea)	+ (flask)	–	3	AN, AT
*Paratetilla* Dendy, 1905	–	+ (hemi-spherical or narrow)	+ (calthrop-like)	5	IP
*Cinachyrella* Wilson, 1925	–	+ (hemi-spherical)	–	42	AT, CA, IP
*Amphitethya* Lendenfeld, 1907	+ (amphiclads)	–	+ (amphiclads)	2	IP
*Fangophilina* Schmidt, 1880	–	+ (differentiated, narrow)	–	4	AT, CA, IP
*Acanthotetilla* Burton, 1959	+ (megacanthoxea)	+ (narrow)	+ (megacanthoxea)	7	AT, CA, IP
*Antarctotetilla* Carella et al., 2016	pseudocortex (oxeas loosely arranged)	–	–	4	AN
*Levantiniella* Carella et al., 2016	–	+ (small, rounded)	–	1	AN

The species *P.bacca* and *C.australiensis* share an obscure taxonomic history, including incomplete descriptions, intermingled identifications, and tens of different species synonymized (see synonyms of *C.australiensis* in Burton 1934: 523, and *P.bacca* in Burton 1959: 200). Therefore, we expected that a detailed revision would reveal species lumped together under both taxonomic entities. The aims of this paper are two-fold: (1) to review the taxonomic history of the genus *Paratetilla* and the species *Cinachyrellaaustraliensis*, and (2) to identify and describe the different *Paratetilla* and *Cinachyrella* species from Indonesia in the Naturalis Biodiversity Center collection.

## Materials and methods

### Taxonomic revision

Literature from 1867 to date was reviewed in order to compile the descriptions of the 11 nominal species for the genus *Paratetilla* Dendy, 1905. The *Cinachyrella* species revision was based on the literature cited by Burton (1934), who lumped together 16 nominal species as synonyms of *Cinachyrellaaustraliensis* (Carter, 1886). The World Porifera Database WPD (van Soest et al. 2018) was used as a valuable guide for consulting the valid species and addressing the literature review. Type material and reference collections deposited at the American Natural History Museum (**AMNH**) in New York, at the Smithsonian Institution National Museum of Natural History (**NMNH**) in Washington D.C., the Natural History Museum (**NHMUK**, formerly **BMNH**) in London, and the Naturalis Biodiversity Center in Leiden (**RMNH**), were examined. The majority of the holotypes were studied for the current research; the ones we did not review were either unavailable or the description of the text was clear and comprehensive.

### Sampling

Individuals of *Cinachyrella* spp. and *Paratetilla* spp. were collected by snorkelling and SCUBA diving during expeditions to Bali (2003), Bunaken (Sulawesi, 2006), Pulau Seribu (Java, 2005), Raja Ampat (Papua, 2007), Berau (East Kalimantan, 2008), and Ternate (Moluccas, 2009). Sampling was systematically achieved in marine habitats such as coral reefs and mangroves, and within marine lakes (Raja Ampat and Berau). Specimens were photographed *in situ* and notes made on morphological and ecological features such as color, size, depth, and substrate. A total of 237 specimens were collected and preserved in ethanol 70%; an additional 11 specimens from the Naturalis Biodiversity Center collection from Indonesia and elsewhere were reviewed as well as 20 type specimens. Table 2 provides an overview of sample numbers per species and Suppl. material 1 (Table S1) provides full collection details per sample.

**Table 2.** Number of samples reviewed per taxon. The column “Indonesia” refers to all samples recently collected in Indonesia (years 2006–2011), “other material” to older specimens in museum collections from Indonesia or other countries; “types” refer to type specimens of valid species and junior synonyms.

Species	Indonesia	Other material	Types	Total
* Paratetilla bacca *	38	4	4	46
* Paratetilla arcifera *	21	4	1	26
* Cinachyrella australiensis *	117	3	9	129
* Cinachyrella porosa *	47	–	5	52
* Cinachyrella paterifera *	14	–	1	15
Total	237	11	20	**268**

### Morphology

Radial and superficial histological sections of sponges were hand cut with a surgical blade; tissue sections were dried on a heat-plate more than 1 hour, mounted in Durcupan ACR resin and examined using light microscopy. Spicule preparations were made by dissociation of a fragment of sponge in sodium hypochlorite and consecutive washing steps, three times in distilled water, twice in 70% ethanol, and suspending in 95% ethanol. The dissociated spicules were dropped onto glass microscope slides, dried and mounted in Durcupan for light microscopy. Spicule preparations for Scanning Electron Microscopy (SEM) were made after two extra washing steps with 95% ethanol. Spicule dimensions and character definitions follow Rützler (1987), Rützler and Smith (1992) and van Soest and Rützler (2002). Spicule dimensions are based on 25 measurements for type specimens and for reference material. Data are given as minimum–mean–maximum in the text.

## Results and discussion

### Systematic descriptions

#### Order Astrophorida

##### Family Tetillidae Sollas, 1888

###### Genus *Paratetilla* Dendy, 1905

The genus *Paratetilla* was established by Dendy (1905) based on the presence of a layer of modified triaenes (calthrops-like). Eleven nominal species have been described with this diagnostic character: *Stellettabacca* Selenka, 1867, *Tethyamerguiensis* Carter, 1883, *Tetillaternatensis* Kieschnick, 1896, *Tetillaamboinensis* Kieschnick, 1898, *Tetillaviolacea* Kieschnick, 1898, *Tetillarubra* Kieschnick, 1898, *Paratetillacineriformis* Dendy, 1905, *Paratetillaeccentrica* Row, 1911, *Paratetillaaruensis* Hentschel, 1912, *Paratetillacorrugata* Dendy, 1922, and *Paratetillalipotriaena* de Laubenfels, 1954. The revision of the taxonomic history of these species reveals that some ambiguous statements have been made (Table 3).

**Table 3.** Historic milestones in the taxonomy of the genus *Paratetilla* Dendy, 1905. Asterisk (*) indicates misidentification of *Cinachyrella* specimens as *Paratetilla*.

Year	Author	Descriptions / Statements
1867	Selenka	Description of *Stellettabacca*. Selenka’s material was collected in Samoa Island and due to the presence of triaenes this species was associated to the family Corticatae (now Astrophorida: Ancorinidae). The description is brief but the sketches included are illustrative, including “Vierstrahler” (=calthrop-like) spicules. Sigma-like spicules are neither mentioned in the description nor drawn in the figures. Currently, type specimen could not be located.
1883	Carter	Description of *Tethyamerguiensis*, including sigmaspires, calthrop-like spicules, oxeas and triaenes and their respective measurements and sketches.
1884	Ridley	In his monograph, Ridley kept *Stellettabacca* in the genus *Stelletta*. The diagnostic characteristic for *Stelletta* for his decision was the absence of bacillar or acerate flesh-spicules. He also noticed that the Samoan *Stelletta* “is probably a *Tethya*, as its stellate agrees with the large stellate of that genus, and its forks are rare and probably foreign to the sponge” (see footnote in Ridley 1884, p. 472).
1887	Vosmaer	Statement about *Stellettabacca* mentioning that it can hardly belong to *Stelletta* genus without further argumentation.
1888	Sollas	Establishment of Family Tetillidae, type genus *Tetilla* Schmidt, 1868. Sponges in this family have sigmaspires (microscleres) and slender protriaenes (megascleres) as diagnostic characters. In this family Sollas included the species Craniella (Alcyonium) craniumMüller (1789), species under the genus *Tethya* by Lamarck (1815) and Gray (1867), and species within the group Tethyina Carter (1875). Carter’s material of *Tethyamerguiensis* was redescribed and transferred to the genus *Tetilla*, as *Tetillamerguiensis*. Tethyacraniumvar.australiensis was redescribed as *Tetilla* (?) *australiensis*. Many other species were also described by Sollas within this family.
1896*	Kieschnick	Description of *Tetillaternatensis* based on material from Ternate Island (Indonesia); he mentioned “Vierstrahler” (=calthrops).
1897	Lindgren	*Tethyamerguiensis* Carter, 1873 as junior synonym to *Stellettabacca*, based on a comment by Sollas (1888, p. 205) of his monograph: “*Stellettabacca*, Selenka, which Vosmaer correctly excludes from *Stelletta*, while Ridley includes it, is as mounted preparations show, identical with *Tetillamerguiensis*, Carter”. However, neither Ridley (1884) nor Vosmaer (1887) supported their inclusion or exclusion of the species with any description of the Selenka specimen, but apparently, they were based merely on the published description. It is remarkable that Sollas in the same monograph (1888) identified the Challenger specimens as *Tetillamerguiensis*, including for the first time this genus and species under the family Tetillidae due to the characteristic sigmaspires.
1898*	Lindgren	Redescription of *Tetillabacca*, with *Tetillamerguiensis* as junior synonym, including material of Torres Straits (North Australia), two localities at Java (Indonesia) and Carter’s specimens from Mergui Archipelago. Size range for each station is shown for oxeas and triaenes, arguing that larger spicules are found to the west while smaller sizes to the east. Redescription of *Tetillaternatensis* based on Java material. It is remarkable that he mentioned the presence of numerous microxeas (240 × 4 µm) and sigmaspires 24 µm.
1898	Kieschnick	Description of *Tetillaamboinensis*, *Tetillaviolacea* and *Tetillarubra* from Amboina Island, all of them with “Vierstrahler” (=calthrop-like) spicules. *T.amboinensis* and *T.violacea* with calthrops in a layer below the surface of the sponge; while the former is characterized by smaller number of triaenes and bundles of oxeas up to the surface of the sponge, the latter by very abundant triaenes, bundles of oxeas projected over the surface of the sponge, and a typical violet color. *T.rubra* separated from the other two by its brick-red color and with calthrops mainly on the basal part of the sponge.
1900	Kieschnick	Extensive description of the same three new species.
1900*	Thiele	Redescription of *Tetillaternatensis* Kieschnick, 1896. Thiele drew attention on the misidentification of *T.ternatensis* by Lindgren (1898), clarifying that Lindgren specimens exhibited microxea resembling *Tetillaaustraliensis* (Carter, 1886). Moreover, Thiele proposed that *T.ternatensis*, as well as Kieschnick’s species *T.amboinensis*, *T.violacea* and *T.rubra*, should be junior synonyms of *T.bacca* arguing that *T.bacca* shows a large morphological variability.
1900*	Kirkpatrick	Extension of the geographical range of *T.bacca* and *T.ternatensis* to Christmas Island. *T.bacca* specimens were described with identical spicules to Lindgren’s (1898) material from Java. *T.ternatensis* also similar to Lindgren’s (1898) material of *T.ternatensis.*
1903*	Lendenfeld	Designation of a new species *Tetillalindgreni* based on Lindgren’s specimens (1898) from Java and Kirkpatrick’s specimens (1900) from Christmas islands, both identified as *T.ternatensis* without calthrops and with small microxeas. Thus, Lendenfeld concluded that those specimens belong to a new species (*T.lindgreni*) because they did not show calthrops as in the original description of Kieschnick (1896). Junior synonyms for *Tetillabacca*, including the material of Selenka, Carter, Sollas, and Kirkpatrick. *T.ternatensis* and *T.violacea* described by Kieschnick and recorded by Thiele (1900) were also included as junior synonyms of *T.bacca*. *Tetillaamboinensis*Kieschnick (1898) was transferred to genus *Cinachyra* and *T.rubra* was established as its junior synonym.
1905	Dendy	The genus *Paratetilla* was erected within the family Tetillidae, based on the presence of calthrop-like spicules. Thus, *Tetillabacca* is transferred to *Paratetilla* genus, including their junior synonyms *T.merguiensis*, as well as the three Kieschnick’s species *T.ternatensis*, *T.amboinensis* and *T.violacea* based on Thiele’s annotation (1903). Description of *Paratetillacineriformis* based on material from Gulf of Manaar (Sri Lanka). Although the spicules shown by *P.cineriformis* resembled *T.merguiensis*, Dendy (1905) argues that the general aspect of the sponge was quite different as porocalices have no specific arrangement and the layer of calthrops was more irregular than in Carter’s species.
1907	Lendenfeld	The genus *Amphytethya* was created based on its characteristic amphitriaenes. Many other species under the genus *Cinachyra*, *Fangophilina* and *Tetilla* were described.
1911	Row	Description of *Paratetillaeccentrica* from the Red Sea. Cortical triaenes (= calthrop-like) with high modifications, in some cases even becoming into “walking-sticks”.
1912	Hentschel	Description of *Paratetillaaruensis* from Aru- and Kei- Islands (Indonesia), with characteristic amphitriaenes. Relocation of the genus *Amphitethya* Lendenfeld, 1907 as a junior synonym of *Paratetilla*.
1922	Dendy	All nominal species with calthrop-like spicules were synonymized to *Paratetillabacca*, except for *P.aruensis* Hentschel, 1912. Two varieties were identified: P.baccavar.violacea based on *T.violacea* characteristics, and the new variety P.baccavar.corrugata from Diego Garcia in the Indian Ocean.
1925	Wilson	Description of *Paratetillaarcifera* from Philippines. Wilson recognized as valid four additional species: *P.bacca* (Selenka, 1867), *P.amboinensis* (Kieschnick, 1898), *P.cineriformis* (Dendy, 1905) and *P.eccentrica* (Row, 1911). However, he also commented that *P.bacca* is a comprehensive variable species, as previously proposed by Thiele (1903) and later established by Dendy (1922). Establishment of *Cinachyrella* genus. Validation of the genus *Amphitethya* Lendenfeld, 1907.
1954	de Laubenfels	Description of *Paratetillalipotriaena* from Micronesia (West-Central Pacific), characterized by variable calthrop-like spicules and the absence of triaenes, and relatively similar to *P.eccentrica* Row, 1911.
1959	Burton	All nominal species described within the genus *Paratetilla* were included as synonyms of *P.bacca*, except for *P.lipotriaena*.
1987	Rützler	Review of Family Tetillidae, including seven genera (all except for *Fangophilina*). Nomination of *Paratetillacineriformis* as type species of genus *Paratetilla*.
1994	Hooper and Wiedenmayer	Review of all *Paratetillabacca* synonyms based on Burton (1959) taxonomic decision.
2002	van Soest and Rützler	Review of the eight genera included within family Tetillidae. Although *Paratetilla* characters were a combination of two descriptions, a paragraph in the discussion included the size differences between both Selenka’s and Carter’s material (*Stellettabacca* and *Tethyamerguiensis*, respectively). The origin of calthrop-like spicules was also discussed as probably modified plagiotriaenes resembling some *Cinachyrella* species, arguing the possibility of the inclusion of the widespread species *Paratetillabacca* within *Cinachyrella* genus.
2008	van Soest and Beglinger	Redescription of *Paratetillacorrugata* based on material from the Gulf of Oman, and giving validity to the variety P.baccavar.corrugata by Dendy (1922). The presence of trichodragmata is characteristic of this species.
2018	van Soest et al. (WPD)	Junior synonyms for *Paratetillabacca* (Selenka, 1867): *Tetillabacca* (Selenka, 1867), *Stellettabaccabacca* Selenka, 1867, *Tethyamerguiensis* Carter, 1883, *Stellettabacca* Selenka, 1887, *Tetillaviolacea* Kieschnick, 1896, *Tetillaternatensis* Kieschnick, 1896, *Tetillarubra* Kieschnick, 1898, *Paratetillacineriformis* Dendy, 1905, *Paratetillaeccentrica* Row, 1911, *Paratetillaarcifera* Wilson, 1925. Other accepted *Paratetilla* species in WPD: *Paratetillaamboinensis* (Kieschnick, 1898), *Paratetillaaruensis* Hentschel, 1912, *Paratetillacorrugata* Dendy, 1922, *Paratetillalipotriaena* de Laubenfels, 1954.
2018	This study	*Paratetilla* species from Indonesia: *Paratetillabacca* (Selenka, 1867), *Paratetillaarcifera* Wilson, 1925, and *Paratetillacorrugata* Dendy, 1922 (not observed in our Indonesian material), *Paratetillaaruensis* Hentschel, 1912 with amphitriaenes, it is suggested to be transferred to *Amphitethya*.

Recent checklists and biodiversity studies in the Indo-Pacific have only recorded *P.bacca*, following Burton’s taxonomic decision in 1959 to synonymize all nominal *Paratetilla* species except *P.lipotriaena*. Two exceptions were found in the literature, the review by Desqueyroux-Faundez (1981) of Topsent’s material (1897) from Amboina Island, who identified it as *Paratetillamerguiensis*, and the inventory of sponges from South China Sea by Hooper et al. (2000), where *P.arcifera* was listed in addition to *P.bacca*.

####### Paratetilla bacca

Taxon classificationAnimaliaTetractinellidaTetillidae

(Selenka, 1867)

Figs 1 , 2

**Figure 1.**
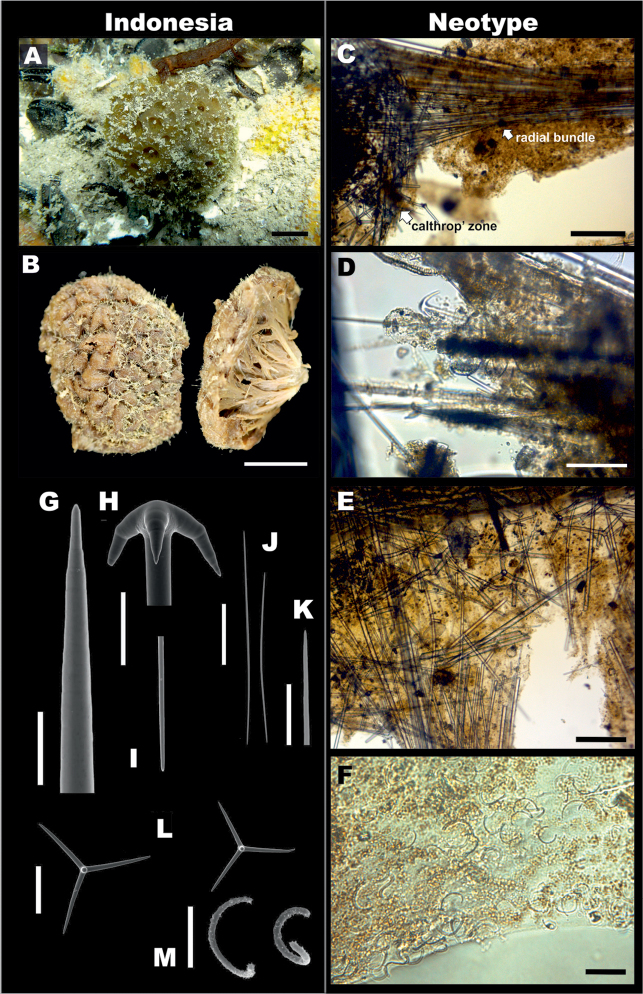
*Paratetillabacca*. **A,B, G-M**RMNH.POR.11292, Kakaban Lake, Indonesia (left side). **C–F** neotype material of *Paratetillabacca*, ZMA.POR.13029, Tutuila Island, American Samoa (right side). **A***in situ* photograph **B** preserved specimen showing the porocalices (scale bar 1 cm) **C** skeleton showing oxeas, calthrops and triaenes **D** skeleton, showing anatriaenes, protriaenes and oxeas **E** skeleton showing detail of the ‘calthrop’ zone **F** sigmaspires **G** oxea, detail **H, I** anatriaene, cladus and rhabd end, **J** thin microxea, **K** thin microxea, detail **L** calthrops **M** sigmaspires. Scale bars: 200 μm (**A–C**); 40 μm (**D, G–I**); 200 μm (**E**); 20 μm (**F**); 50 μm (**J**); 10 μm (**K, M**); 100 μm (**L**).

Stelletta bacca Selenka, 1867: 569, pl. xxxv, figs 14, 15 (type not found, material from type locality seen).
Tethya merguiensis Carter, 1883: 366, pl. xv, figs 6–8; Carter, 1887: 80 (type seen).
Tetilla merguiensis ; Sollas, 1888: 14; Topsent, 1897: 441, pl. xviii, fig. 4–5, pl. xxi figs 34.
Tetilla ternatensis Kieschnick, 1896: 527. Thiele, 1900: 39, pl. ii, fig 13; Not Tetillaternatensis Lindgren, 1898: 329 pl. 17, fig. 14; pl. 19, Fig. 25 a-e, a’, b’.
Tetilla bacca ; Lindgren, 1897: 485; Lindgren, 1898: 328; Thiele, 1900: 39, pl. ii, fig 13; Kirkpatrick, 1900: 132 (material seen); Lendenfeld, 1903: 19.
Tetilla amboinensis Kieschnick, 1898: 10.
Tetilla violacea Kieschnick, 1898: 15.
Tetilla rubra Kieschnick, 1898: 18.
Paratetilla cineriformis Dendy, 1905: 97, pl. iii, fig. 7 (type seen).
Paratetilla eccentrica Row, 1911: 306, pl. xxxv, fig. 1, pI. xxxvi, fig. 8 (type seen).
Cinachyra amboinensis ; Hentschel, 1912: 331.
Paratetilla bacca ; Dendy, 1922: 21 (material seen).
Paratetilla bacca var. violacea ; Dendy, 1922: 22, pl. 1, fig. 6 (material seen).
Paratetilla lipotriaena de Laubenfels, 1954: 244, text figure no. 168 (type seen).

######## Material examined.

Neotype ZMA.POR.13029, Tutuila Island, American Samoa. Holotype of first junior synonym *Tethyamerguiensis* Carter, 1883 (?) NHMUK 1894.11.16.17, Mergui Archipelago, Myanmar. Holotype NHMUK 1954.2.23.106 Gulf of Manaar, Sri Lanka (as *Paratetillacineriformis* Dendy, 1905). NHMUK unreg. type, Crossland Collection, Red Sea (as *Paratetillaeccentrica* Row, 1911). NHMUK 1898.12.20.19, Flying Cove Fish, Christmas Islands (as *Tetillabacca*=*Paratetillamerguiensis* Kirkpatrick, 1900). NHMUK 1921.11.7.10, Sealark Sponges, Indian Ocean (as Paratetillabaccavar.violacea). Holotype USNM 23049, East part of Lagoon, Ponape, Caroline Islands, 1 Aug 1949 (as *Paratetillalipotriaena* de Laubenfels, 1954). **INDONESIA**. Bali, *Bali reef*, RMNH.POR.1732; East Kalimantan, *Berau reef*, RMNH.POR.11281, RMNH.POR.11282, RMNH.POR.11283; *Kakaban Lake*, RMNH.POR.11289, RMNH.POR.11290, RMNH.POR.11291, RMNH.POR.11292, *Haji Buang Lake*, RMNH.POR.11284, RMNH.POR.11287, RMNH.POR.11288, RMNH.POR.11285, RMNH.POR.11286, RMNH.POR.3515. Sulawesi, *Bunaken reef*, RMNH.POR.3100, RMNH.POR.3106, RMNH.POR.3115; *Bunaken mangrove*, RMNH.POR.2819; *Spermonde Archipelago*, ZMA.POR.13221. Ternate, *Ternate reef*, RMNH.POR.5344, RMNH.POR.5467. West Papua, *Wallace Lake*, RMNH.POR.11293, RMNH.POR.11294, RMNH.POR.11295; *Outside Wallace Lake*, RMNH.POR.11296, RMNH.POR.11297, RMNH.POR.11298; *Ctenophore Lake*, RMNH.POR.11302; *Gam Mangrove*, RMNH.POR.11299, RMNH.POR.11300, RMNH.POR.11301; *Outside Ctenophore Lake*, RMNH.POR.11303; *Big Caulerpa Lake*, RMNH.POR.11304; *Gam Island*, RMNH.POR.11305, RMNH.POR.11306, RMNH.POR.11307.

**Other material**: East Kalimantan, *Makassar Straits*, ZMA.POR.1735, Siboga Expedition, St. 81. Singapore, RMNH.POR.2506, RMNH.POR.2512. Western Indian Ocean, ZMA.POR.20673.

######## Description.

***External morphology.*** Globular sponges, size between 1 and 5 cm in diameter. Surface hispid due to the projecting spicules, covered by numerous porocalices (Figure 1A, B). Porocalices are bowl-shape, with oval to circular apertures, up to 5 mm in diameter and 7 mm deep, numerous, scattered uniformly over the surface of the sponge; in preserved material, some porocalices are closed and only a narrow aperture is visible giving to the sponge a rough appearance. External color generally brown when alive, which turns dark brown in ethanol, choanosome light brown, and has a ‘dried out’ appearance (Figure 1B). Numerous small dark brown granules in the tissue (Figure 1E, F). Consistency compact.

**Skeleton.** No cortex. Choanosomal skeleton composed by bundles of oxeas and triaenes radiating from a central core, ⅕–⅓ of the diameter of the sponge.

**Megascleres.** The material from Indonesia and the type of *P.merguiensis* have oxeas 850–3085.3–4500 mm × 5–41.5–65 mm (Table 4, Figure 1E, D, G). Anatriaenes always present, very abundant, cladi stout, slightly flattened, 20–62.6–100 mm × 12.5–48.3–75 mm, long rhabd up to 6000 × 20 mm, tapering to dimensions much less than 1mm (Figure 1H, I). Protriaenes scarce in some specimens and absent in the type specimen; when present, they exhibit two different shapes, the first one with stouter and smaller cladi, the second one with thinner and larger cladi (27.5–53.9–100 mm × 37.5–107.4–200 mm × 2.5–6.5–12.5 mm), rhabd up to 5850 × 15 mm, tapering to dimensions of < 1 mm. Calthrop-like short shafted triaenes, three types are distinguished with a wide range of sizes, from which measurements are shown as a general summary (Table 4). In the first type, four rays can be recognized (Figure 1L), three of them large, up to 400–600 mm, and a short one up to 100 mm long, usually pointing down to the centre of the body; the second one with three rays, almost the same length up to 400 mm; and the third one with three rays as well, two of them in an angle of 180° and the other one perpendicular, 50–100 mm. The calthrops are located immediately below the surface, constituting more or less a homogeneous layer.

**Table 4.** Spicule measurements of six specimens of *Paratetillabacca* and five specimens of *P.arcifera* from different regions (n = 10 per spicule type and dimension with minimum-**mean**-maximum). Asterisk (*) indicate that rhabds of spicules were broken and no measurement was possible.

Measurements		* Paratetilla bacca *						* Paratetilla arcifera *				
		NHMUK94. 11.16.17/16	RMNH.POR.11292	RMNH.POR.11287	RMNH.POR.11281	RMNH.POR.11301	RMNH.POR.5344	USNM21278 (Holotype)	RMNH.POR.11266	RMNH.POR.11273	RMNH.POR.11310	RMNH.POR.3114
**Locality**		Mergui Archipelago	Kakaban Lake	Haji Buang Lake	Berau	Raja Ampat	Ternate	Philippines	Berau	Berau	Ternate	Manado
**Habitat**		Reef	Marine Lake	Marine Lake	Reef	Mangrove	Reef	Reef	Reef	Reef	Reef	Reef
**Oxeas**	Length	3114.36–**3114.6**–3115	850–**2340.8**–3150	1000–2922–3850	2520–**3324.6**–3850	3100–3270–3500	1250–3540–4500	1650–2435–3125	1650–3093–4500	1600–3041–4175	840–1996–3100	3100–3600–4000
	Width	40.8–**42.5**–51	5–**29.9**–40	12.5–36–50	30–**48.8**–60	30–42–55	25–49.6–65	20–39.5–65	25–42.2–55	20–33.7–50	10–25.4–50	27.5–43–52.5
**Anatriaenes**	Rhabd length	*	3000–**3677.8**–4600	3900–4741.7–5300	4250–**5057.1**–6000	*	*	*	*	*	*	*
	Rhabd width	15–**16**–20	5–**12.9**–20	12.5–**14.8**–17.5	7.5–**10.7**–15	7.5–11.3–15	5–7.5–10	*	*	*	5–5.7–7.5	5–6.3–7.5
	Cladi total	20–**27.9**–40	37.5–**58.7**–75	80–**91.5**–100	40–73.6–90	60–74.5–80	22.5–49.5–75	40–68–80	60–65.6–80	22.5–39.8–60	40–48.2–50	50–65–75
	Cladi length	40.8–**52**–71.4	25–**44.4**–65	50–**63.9**–75	20–40.5–50	40–57.3–75	12.5–31.8–50	25–39.4–45	30–38.7–47.5	10–21.3–30	15–20.9–25	35–40.6–50
	Cladi width	10–**12**–15	7.5–**12.1**–20	7.5–**10.9**–15	7.5–10.7–15	7.5–9.5–10	5–7-7.5	5–8.2–10	5–6.6–10	2.5–5-7.5	5–5-5	5–6.3–7.5
**Protriaenes**	Rhabd length	*	3900–**3900**–3900	3000–**4434.6**–5850	3100–3800–4500	*	*	*	*	*	*	*
	Rhabd width	*	7.5–**10**–12.5	10–**13.3**–17.5	5–8.6–15	2.5–4.8–5	5–5-5	*	*	*	*	5–6-7.5
	Cladi total	*	27.5–**46.9**–70	40–**70.7**–100	50–67.9–100	30–54–70	30–30–30	*	30–38.3–40	40–40–40	*	30–42.8–50
	Cladi length	*	47.5–**84.4**–100	85–**140.5**–185	110–141.4–200	50–133–170	37.5–37.5–37.5	*	50–61.7–70	75–75–75	*	25–40.3–60
	Cladi width	*	5–**7.5**–10	7.5–**9.5**–12.5	5–7.9–10	2.5–2.5–2.5	5–5-5	*	2.5–5.4–7.5	5–5-5	*	2.5–4.3–5
**Calthrops**	C1	42.5–**168.1**–255	110–**266**–475	270–**369.8**–510	140–296.7–360	220–301–350	250–375.7–600	320–362.5–430	150–253.9–375	110–154–220	150–192.5–250	120–179–220
	C2	22.5–**92.9**–183.6	90–**225**–325	220–**346.4**–460	140–281–350	210–284–350	240–291.4–350	230–287.5–320	75–239.4–390	90–134.4–160	70–125–230	90–129–200
	C3	20–**106**–234.6	40–**203.7**–325	50–**292.5**–400	25–218.7–345	180–254–310	200–272.9–350	120–195–300	140–245.5–355	60–110–150	50–93.3–160	90–129–200
	Width	3.5–**12.2**–20.4	7.5–**18.3**–35	15–**31.7**–45	10–18.3–27.5	10–14.5–17.5	17.5–20.4–25	15–18.8–22.5	10–18.3–25	10–12.5–15	10–13.8–17.5	12.5–15–20
**Microxea**		173.4–**195.3**–224.4	105–**136.2**–212.5	170–**213.6**–250	250–316.8–385	210–264–300	250–323.6–380	180–308.4–380	270–323.2–400	200–342–500	340–370–410	250–367–450
**Sigmaspires**		12.5–**14.4**–17.5	10–**13**–17.5	12.5–**15.4**–25	12.5–14.2–17.5	12.5–13.8–15	12.5–14–17.5	7.5–12.5–17.5	12.5–15.4–17.5	15–16.3–20	12.5–15.3–17.5	12.5–14–17.5

**Microscleres.** Thin microxeas are common, 105–241.6–380 mm, ‘hair-like’. Sigmaspires, 10–14.1–25 mm, C-S shape (Figure 1F, M).

######## Ecology.

Inhabiting all studied environments in Indonesia, including coral reefs, mangroves, and marine lakes. Specimens more common in mangroves and marine lakes, and shallow reef flats where they are usually found on dead coral skeletons or coral rubble, typically ranging in depth from 0–5m. No specimens collected from deeper coral reefs in Indonesia.

######## Distribution.

**Figure 2.**
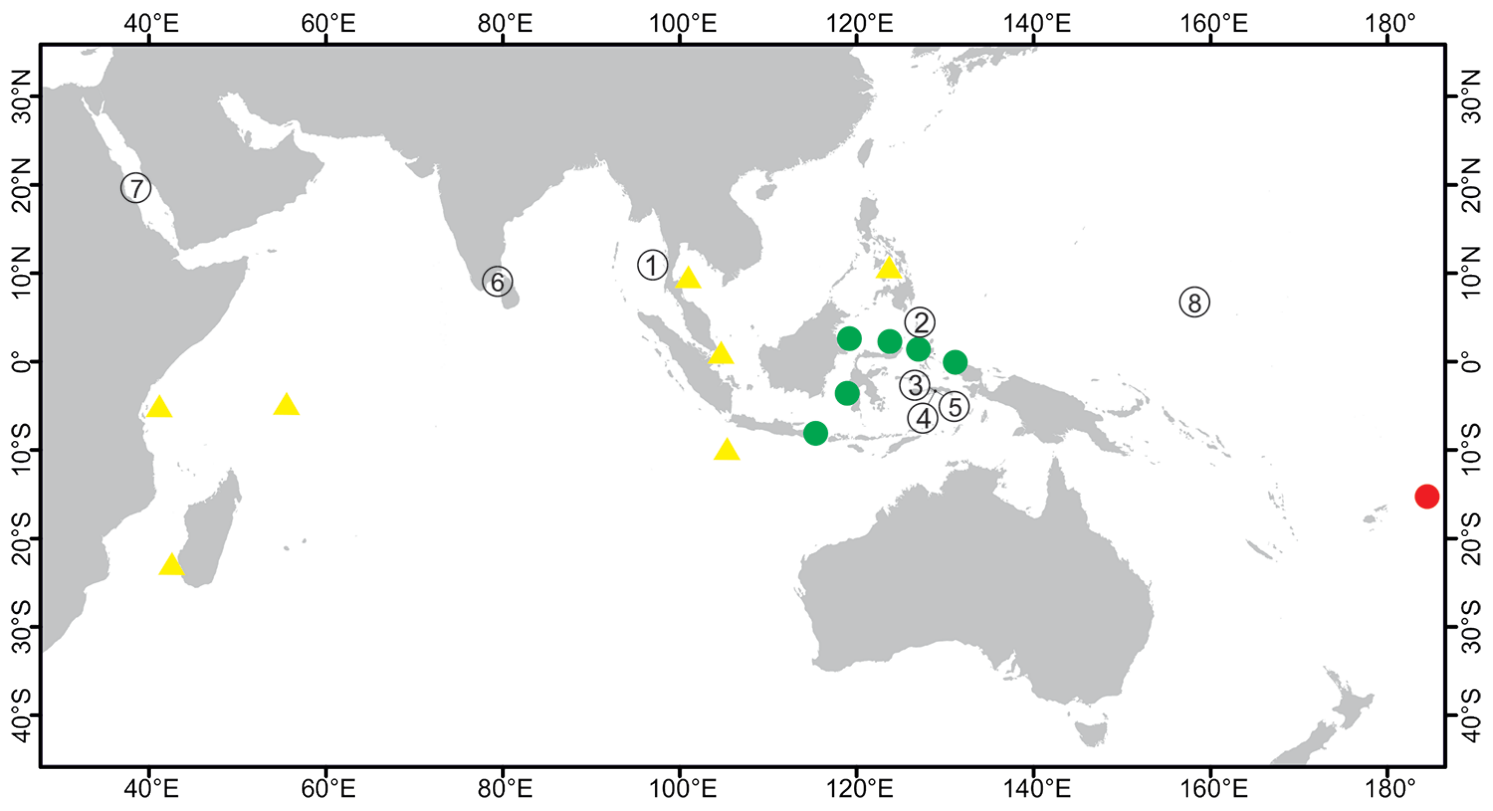
Distribution of *Paratetillabacca*. Red dot: type locality, *Stellettabacca* Selenka, 1867, American Samoa. Green dots: Indonesian localities where the species was collected recently. Yellow triangles: Records from localities outside Indonesia, Zanzibar, Southwest Madagascar, Seychelles, Thailand, Singapore, Christmas Island, and Philippines. Circled numbers: type localities of synonymized species, **1***Tethyamerguiensis* Carter, 1873, Mergui Archipelago **2***Tetillaternatensis* Kieschnick, 1896, Ternate Island **3***Tetillaamboinensis* Kieschnick, 1898, Ambon Island **4***Tetillaviolacea* Kieschnick, 1898, Ambon Island **5***Tetillarubra* Kieschnick, 1898, Ambon Island **6***Paratetillacineriformis* Dendy, 1905, Gulf of Manaar, Sri Lanka. **7***Paratetillaeccentrica* Row, 1911, Tella Tella Kabira, Red Sea **8***Paratetillalipotriaena* de Laubenfels, 1954, Matalanim, Eastern Pohnpei, Micronesia.

*Paratetillabacca* has a wide distribution in Indonesia, including Berau, Bunaken, Raja Ampat, Ternate, and Java. Previous Indonesian records are from Spermonde Archipelago (Becking et al. 2006), Berau (de Voogd et al. 2009), and Raja Ampat (Becking 2008). In addition, this species has also been reported from Seychelles Islands (Thomas 1973), Southwest Madagascar (Vacelet et al. 1976), Zanzibar (Pulitzer-Finali 1993), Thailand (Putchakarn 2007), Singapore (Lim et al. 2008), Philippines (Longakit et al. 2005) (Figure 2).

######## Remarks.

We did not succeed in locating the holotype of *Paratetillabacca*, despite concerted effort. At this time, we assume that the type is no longer available. The description by Selenka of the type specimen does not mention the occurrence of any type of sigmaspires. It is a matter of speculation whether Lindgren (1897) actually examined Selenka’s material to propose Carter’s species *Paratetillamerguensis* as a junior synonym to *Paratetillabacca*, or whether he based his conclusion merely on the literature. It is possible that sigmaspires may have been overlooked by Selenka in his original description and drawings, yet the arrangement of the megascleres in the skeleton shows a clear similarity with Carter’s species *P.merguensis* (Suppl. material 2, Figure S1). In contrast to Selenka’s description, Carter (1883) included a complete and detailed account of *P.merguiensis*, which was verified through examination of two slides deposited in the NHM collection (NHMUK 1894.11.16–17); few oxeas are complete in these slides (most broken), therefore limited variation of this character was observed. For most of spicule types enough measurements were possible. Although we did not succeed finding Selenka’s type, we did examine one specimen and its associated slide preparation from Samoa identified as *P.bacca* (ZMA.POR.13029), which has all the characteristic spicules, including sigmaspires, that are present in our specimens from Indonesia (Figure 1C-F). This material is designated here with the status of neotype following the rules of the International Code of Zoological Nomenclature, article 75. Therefore, we conclude that *P.bacca* is a valid species, and subsequent species should be designed as junior synonyms. In all of our *Paratetilla* samples, we have furthermore not encountered one specimen without sigmaspires. Here, we show the measurements of the holotype of *P.merguiensis*, as well as specimens from different localities in Indonesia (Table 4). Although there is a large variation in spicules sizes among the different localities, there was also great intra-specific variation and we did not find any reason to declare the validity of any junior synonym included in this revision. In general, populations from marine lakes (Kakaban and Haji Buang) exhibit smaller spicules in comparison with their reefal counterparts at the same localities (Table 4). This variation could be a response to different environmental conditions within the marine lakes (Becking et al. 2011), or a consequence of genetic selection after isolation of these populations about 8000–10000 years ago (Dawson and Hamner 2005, Becking et al. 2013, Becking et al. 2016), or a synergistic effect between environmental and genetic factors.

According to the WPD (van Soest et al. 2018), other four valid *Paratetilla* species are *P.amboinensis* (Kieschnick, 1898), *P.lipotriaena* de Laubenfels, 1954, *P.corrugata* Dendy, 1922 and *P.aruensis* Hentschel, 1912. Based on the description of *P.amboinensis* (Kieschnick, 1898), the shape and skeleton features exhibited by this species fit within the current diagnosis of *P.bacca*, therefore we recommend that these two species should be synonymized. The species *P.lipotriaena* was erected by de Laubenfels based on the absence of triaenes. Our examination of the type specimen (USNM 23049) revealed the presence of triaenes and the same characters as *P.bacca*, therefore we have synonymized this species with *P.bacca.* On the other hand, *P.bacca* can be distingished from *P.corrugata* Dendy, 1922, because of the abundant trichodragmata exhibited by the latter species. Consequently, *P.corrugata* can still be considered a valid species. Finally, the status of *P.aruensis* Hentschel, 1912 within this genus should be reconsidered. After examination of two slides available at the NHMUK, no calthrops were found, only the typical amphitriaenes originally described for this species. Amphitriaenes make this species more similar to the genus *Amphitethya* instead of *Paratetilla*. Further examination of specimens would corroborate our preliminary conclusion.

In a molecular phylogenetic study, which was based in part on specimens that we review in the current study (see Suppl. material 1, Table S1 for corresponding GenBank numbers), Schuster et al. (2017) distinguishes *P.bacca* as a monophyletic clade in the Tetillidae. Due to the wide distribution of this species and large intraspecific morphological variability we recommend further molecular studies, particularly of *P.bacca* from its type locality (American Samoa). This would allow a more detailed description of the genetic variation of *P.bacca* and verify our initial taxonomic proposal based on morphology.

####### Paratetilla arcifera

Taxon classificationAnimaliaTetractinellidaTetillidae

Wilson, 1925

Figs 3 , 4 , 5

Paratetilla arcifera Wilson, 1925: 380; plate 40, fig. 2; plate 48, fig. 6 (type seen).

######## Material examined.

Holotype USNM 21278, Albatross Stn. 5400, Malapascua Island, Cebu, Philippines, 46 m, 16 Mar 1909. **INDONESIA**. East Kalimantan, *Berau reef*, RMNH.POR.11131, RMNH.POR.11265, RMNH.POR.11266, RMNH.POR.11269, RMNH.POR.11267, RMNH.POR.11268, RMNH.POR.11270, RMNH.POR.11271, RMNH.POR.11272, RMNH.POR.11273. Bali, RMNH.POR.1870. Java, *Thousand Islands*, RMNH.POR. 2076. Sulawesi, *Bunaken*, RMNH.POR.3114; *Manado*RMNH.POR.3114. Ternate, *Ternate reef, RMNH.POR.*11310. West Papua, *Kerupiar Island reef*, RMNH.POR.11280; *Outside Ctenophore Lake*, RMNH.POR.11275; *Gam Island*, RMNH.POR.11277, RMNH.POR.11278, RMNH.POR.11279, RMNH.POR.11274, RMNH.POR.11276. **TAIWAN**. *Reef*, RMNH.POR.3196, RMNH.POR.3206, RMNH.POR.3225, RMNH.POR.3236.

######## Description.

**Figure 3.**
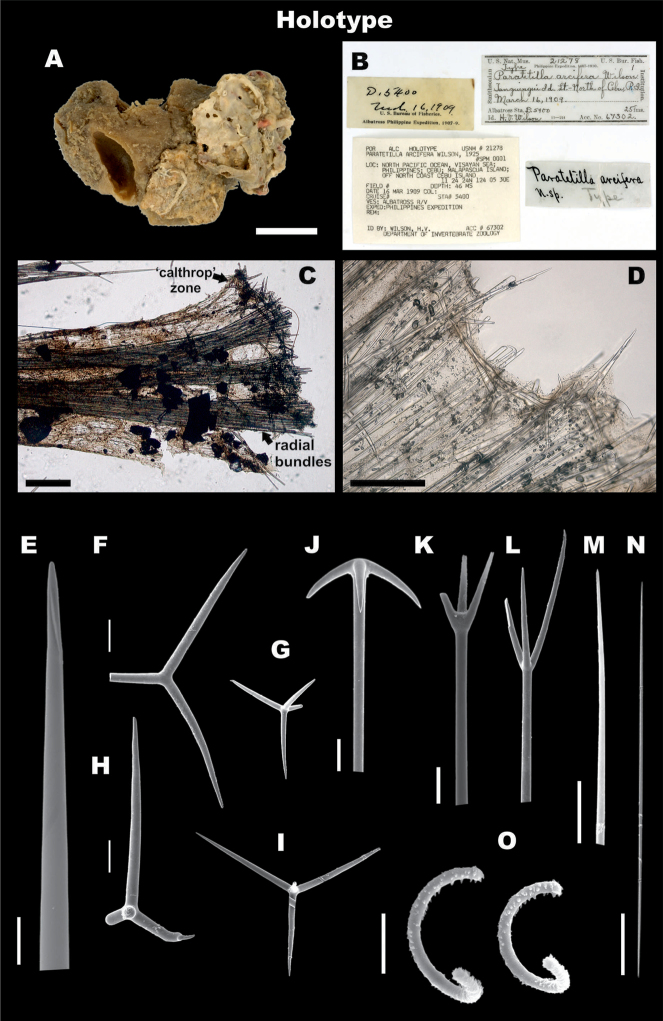
*Paratetillaarcifera*. Holotype USNM 21278, Malapascua Island, Cebu, Philippines **A** preserved specimen showing large porocalices **B** Labels of holotype **C** skeleton, showing calthrops and radial bundles **D** skeleton, showing oxeas, calthrops, and anatriaenes **E** oxea, end detail **F-I** different calthrop shapes and sizes **J** anatriaene **K, L** protriaene, different types **M** thin microxea, detail **N** thin microxea, full length **O** sigmaspires. Scale bars: 1 cm (**A**); 500 μm (**C, D**); 100 μm (**E**); 50 μm (**F–I, N**); 20 μm (**J**); 40 μm (**K, L**); 5 μm (**M, O**).

**Figure 4.**
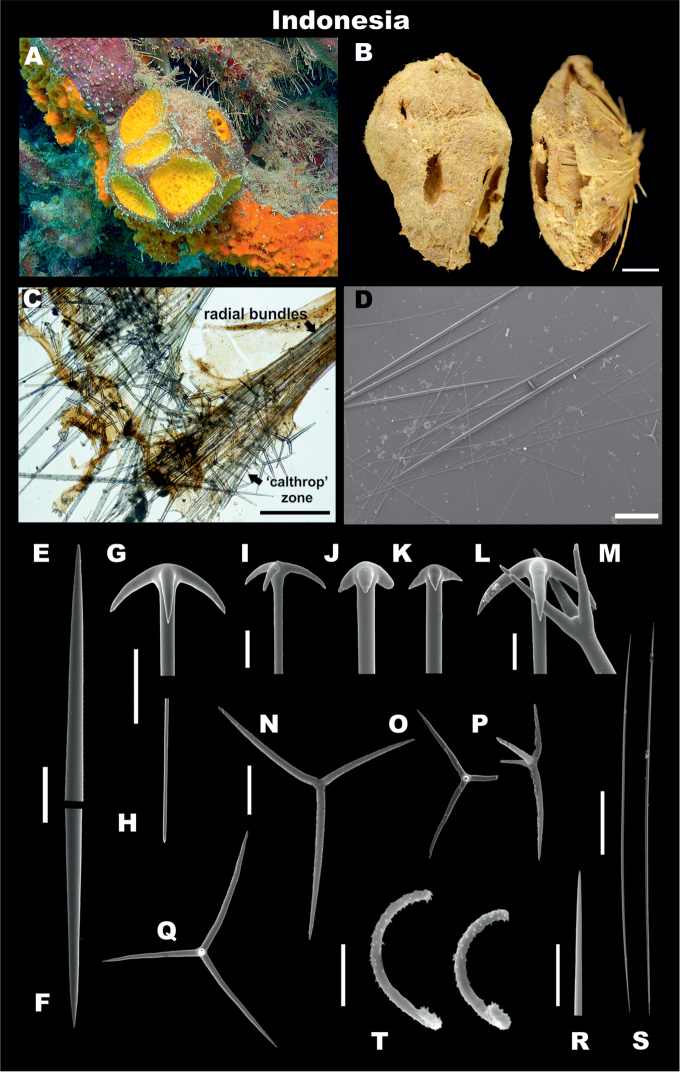
*Paratetillaarcifera* from Indonesia RMNH.POR.11266. **A** in situ photograph. **B** preserved specimen showing the porocalices (scale bar 1 cm) **C** skeleton **D** spicules **E, F** oxea, end detail **G, H** natriaene, cladus. and rhabd end **I-L** anatriaene, different types **M** Protriaene **N-Q** different calthrops **R** thin microxea, detail **S** thin microxeas, full length **T** sigmaspires. Scale bars: 1 cm (**B**), 500 μm (**C,D**), 100 μm **E,F**); 40 μm (**G,H**); 20 μm (**I–M**); 200 μm (**N–Q**); 5 μm (**R, T**); 50 μm (**S**).

**External morphology.** Globular sponges, size from 3 to 6 cm in diameter (Figs 3A, 4A). Surface hispid due to the projecting spicules, covered by numerous porocalices. Porocalices are bowl-shape, with oval apertures, up to 10 × 5 mm and 6 mm deep, few, mainly on the top surface of the sponge; in preserved material, most porocalices remained open (Figs 3A, 4A). Color generally bright orange when alive, which turns darker or even brown in ethanol. No granules in choanosome. Fleshy consistency.

**Skeleton.** No cortex. Skeleton composed by bundles of oxeas and triaenes radiating from a central core, and spaced between each other, giving a softer consistency (Figs 3C, D, 4C).

**Megascleres.** Holotype and Indonesian specimen size ranges are summarized in Table 4. Holotype: Oxeas 1650–2435–4500 mm × 20–36.8–65 mm; anatriaenes very abundant (Figure 3J), rhabds generally broken, up to 6000 × 10 mm, apparently tapering to dimensions of < 1 mm, cladi thin, slightly flattened, 40–68–80 mm × 25–39.4–45 mm × 5–8.2–10 mm; few protriaenes (Figure 3K,L), thinner and small cladi (40–65–80 mm × 60–85–110 mm), rhabds mostly broken, up to 5000 × 15 mm, tapering to dimensions of < 1 mm; two types of calthrop-like short shafted triaenes, one type with four rays of which three are short (150–300 mm) and one is large (400 mm) (Figure 3H), the other type has three rays of almost equal length up to 400 mm (Figure 3 F-G, I); calthrops are abundant in some specimens, but can be in very low numbers till almost absent in some others, they are located immediately below the surface, constituting a thin layer that can be missed in some spicule preparations.

**Microscleres.** Thin microxeas are common, 180–308.4–380 mm, ‘hair-like’ (Figs 3M, N, 4R, S). Sigmaspires, 7.5–12.5–17.5 mm, C-S shape (Figs 3O, 4T).

######## Ecology.

Coral reef habitats at depths from 1- 20/30 m. Absent from marine lakes, mangroves and other localities with higher sedimentation and/or variable salinity.

######## Distribution.

**Figure 5.**
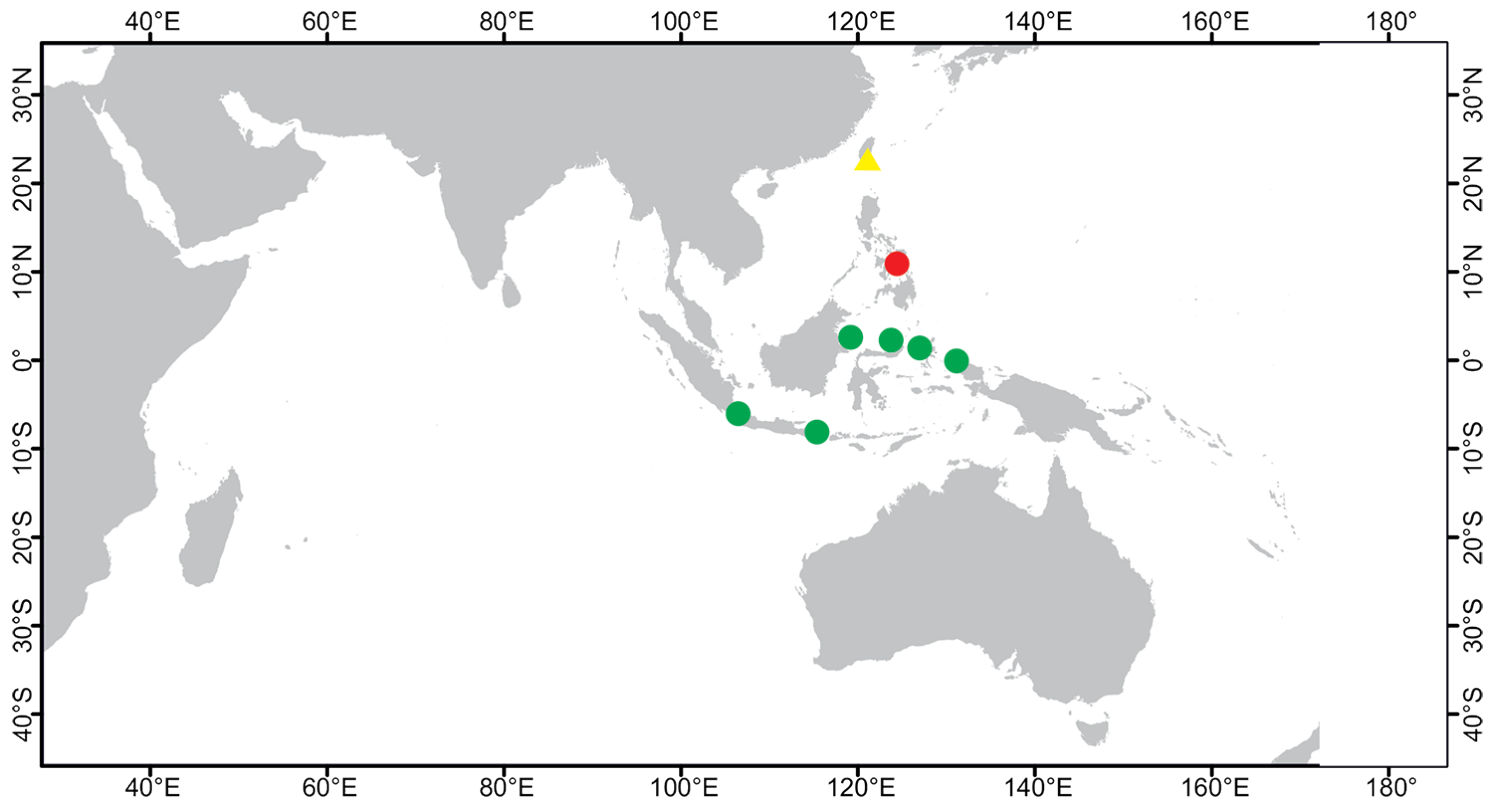
Distribution of *Paratetillaarcifera*. Red dot: type locality, *Paratetillaarcifera* Wilson, 1925, Tanguingui Island, Philippines. Green dots: Indonesian localities where the species was collected recently. Yellow triangle: Records from localities outside Indonesia, Taiwan.

Occur in coral reefs of Berau, Bunaken, Ternate, and Raja Ampat. An additional record from its type locality, Philippines (Wilson, 1925) could be inferred from the literature (see Longakit et al. 2005: Figure 9 as *P.bacca*), and collections from Taiwan (Figure 5).

######## Remarks.

Spicule sizes for most Indonesian specimens vary within the holotype ranges, except for the Ternate population, which exhibits smaller sizes and lack of protriaenes (Table 4). The typical orange color and ‘fleshy’ soft consistency are easy distinctive characters of this species (Figure 4A). The differences between *P.arcifera* and its congener *P.bacca* lie in the stark orange coloring, the fleshy consistency, the lack of granules, the larger porocalices, and thin microxeas generally longer than in *P.bacca*. *P.arcifera* specimens are typically larger than *P.bacca.* We, furthermore, deem *P.arcifera* a distinct species from *P.bacca*, based on recent molecular phylogenetic analyses that included *P.arcifera* (genbank accession number LT628349) and *P.bacca* (LT628350) specimens reviewed in our current study and support the hypothesis of two species (Schuster et al. 2017).

###### Genus *Cinachyrella* Wilson, 1925

Currently, 42 species are valid within the genus *Cinachyrella* according to the WPD (van Soest et al. 2018), including the homonyms of *C.globulosa* and one additional description of *C.cavernosa* (Lamarck, 1815) sensu Burton (1959). Originally, Wilson (1925) grouped certain species of the genera *Tetilla* and Cinachyraunder thesubgenusCinachyrella based on the presence of porocalices (poriferous pits) and the absence of cortex. Subsequently, a complete review of Caribbean species by Rützler and Smith (1992) included four valid *Cinachyrella* species and it was recently complemented with the description of two new species from Brazilian deep waters (Fernández et al. 2018). The most recent review of the Indo-Pacific species was attempted by Burton (1934). In his monograph, Burton established that 16 nominal species were synonyms of the widespread and variable species *Cinachyrellaaustraliensis* (Carter, 1886) (see Table 5). However, the validity of Burton’s conclusion was not accepted by van Soest and Rützler (2002) in the Systema Porifera. Therefore, a further examination of the junior synonyms proposed by Burton (1934) was needed and became one of the principal aims that guide this revision. A general review of the historic events about species descriptions and synonyms is provided in Table 5. Emphasis was given to species described based on Indo-Pacific specimens. Remarks were added to clarify the early confusion introduced by Lindgren (1898) when he identified some *Cinachyrella* specimens as *Tetillaternatensis* (=*Paratetillabacca*), although his specimens have conspicuos acanthose microxea and lack of calthrop-like spicules, misleading later descriptions for both genera.

**Table 5.** Historic milestones in the taxonomy of *Cinachyrellaaustraliensis* and other *Cinachyrella* species from Indonesia. Asterisk (*) indicates misidentification of *Cinachyrella* specimens as *Paratetilla*.

Year	Author	Descriptions / Statements
1873	Gray	Description of the monotypic genera *Psetalia* and *Labaria*, with the species *P.globulosa* and *L.hemisphaerica*, respectively.
1886	Carter	Description of Tethyacraniumvar.australiensis from Port Phillip Heads (South Australia) collected at 36 m depth. This species was characterized by the presence of minutely spined (= acanthose) microxea (210 μm).
1888	Sollas	Establishment of Family Tetillidae. Tethyacraniumvar.australiensis was redescribed as *Tetilla* (?) *australiensis*. In addition, Sollas noted that the characteristic microxeas of *T.australiensis* were also present in *T.merguiensis* as well, but were more abundant in *T.australiensis*.
1888	Lendenfeld	Description of genus *Spiretta* within Family Tetillidae, including two new species *S.raphidiophora* and *S.porosa*, from Port Jackson (SE Australia) and Port Denison (NE Australia), respectively. The former with microxea (240 × 2 µm) and the latter without them.
1891	Keller	Description of *Cinachyraschulzei* from the Red Sea and Mozambique, with microxea 250 × 5 µm.
1896*	Kieschnick	Description of *Tetillaternatensis* based on material from Ternate Island (Indonesia). He mentioned “Vierstrahler” (= calthrops).
1898*	Lindgren	Redescription of *Tetillaternatensis* based on Java material. It is remarkable that he mentioned the presence of numerous microxea (240 × 4 µm) and sigmaspires 24 µm.
1898	Kieschnick	Description of *Tetillaschulzei* from material of NE Australia to Ambon Island, with microxea (198-220 µm × 4 µm). *T.schulzei* has ‘oscula’ that we interpret as porocalices. Although Kieschnick entitled *T.schulzei* as new species, it is not clear if he was aware of *Cinachyraschulzei* described by Keller (1891). Three other *Tetilla* species with “Vierstrahler” (= calthrops) spicules were described (see Table 3).
1899	Thiele	Record of *Tetillaaustraliensis* from Sulawesi (Indonesia). Specimens with acanthose microxea (180-200 µm × 2.5 µm).
1900*	Thiele	With the redescription of *Tetillaternatensis* Kieschnick, 1896, Thiele noticed the misidentification of *T.ternatensis* by Lindgren (1898) and pointed out that Lindgren specimens exhibited microxea resembling *Tetillaaustraliensis* (Carter, 1886).
1900	Kieschnick	Additional record of *Tetillaschulzei* from Ambon Islands, including description of the specimens, with microxea from 198 to 220 µm × 4 µm.
1900*	Kirkpatrick	Extension of geographical range of *T.bacca* and *T.ternatensis* to Christmas Island. *T.bacca* specimens were described with identical spicules to Lindgren’s material from Java. *T.ternatensis* also similar to Lindgren’s material of *T.ternatensis*, this is having microxeas and missing calthrops.
1902	Sollas	Description of *Cinachyramalaccensis* from Malaysia. Cup-shaped porocalices are described together with different spicules, except for microxea. In the available figures, no microxeas are shown.
1903*	Thiele	Redescription of *Tetillaternatensis* Kieschnick, 1898. He drew attention on the misidentification of *T.ternatensis* by Lindgren (1898), clarifying that Lindgren specimens exhibited microxea resembling *Tetillaaustraliensis* (Carter, 1886).
1903*	Lendenfeld	Designation of a new species *Tetillalindgreni* based on *T.ternatensis* material described by Lindgren (1898) and Kirkpatrick (1900), excluding the original description of Kieschnick (1896), because the latter one has calthrop-like spicules. Two *Spiretta* species, *S.raphidiophora* and *S.porosa*, transferred to genus *Tetilla*.
1905	Dendy	Monograph on sponges from Sri Lanka. Description of *Tetillaanomala*, showing remarkable siliceous micro-spherules (4 µm) and no microxeas. Description of *Tetillapoculifera* with smooth microxeas (230 × 5 µm). Description of *Tetillalimicola*, pink-color and root tuft; neither porocalices nor microxea are described. The genus *Paratetilla* was established.
1906	Baer	Description of *Tethyaarmata* from Zanzibar (Africa, Indian Ocean). It is characterized by a dermal cortex formed by microxea (166-296 µm × 1-2 µm).
1907	Lendenfeld	Description of *Cinachyraisis* and *Tethyahebes* from NW Australia, the first one exhibiting smaller microxea (130-160 µm × 2-5.5 µm), and the second one larger rough microxea (= acanthose microxea, 250-275 µm × 4-6 µm). Description of *Cinachyraalba-tridens*, *C.alba-obtusa*, and *C.alba-bidens* species, slightly differentiated by the geometry and abundance of triaenes. He kept the three species because they were collected in three distant localities, Chagos Archipelago, Papua New Guinea, and Tonga Islands, respectively; “alba-group” species do not contain microxeas, and sigmaspires are small (<10 µm).
1911	Row	Description of *Chrotellaibis* from the Red Sea. Species with smooth microxea (150 × 2.1 µm), sharing this character with *Tetillapoculifera*, and *Paratetilla* species *P.merguiensis*, *P.eccentrica* and *P.cineriformis*. In his description, Row clearly differentiated his species from *T.australiensis* due to the latter having acanthose microxea.
1911	Hentschel	Description of *Tetillacinachyroides* from South Australia. Species with acanthose microxea (112-168 µm × 2.5 µm), sigmaspires (10-12 µm) and spherules (5 µm).
1912	Hentschel	Description of *Cinachyramertoni* and *Cinachyranuda* from Aru- and Kei- Islands (Indonesia). Both species contain microxea, the first one smooth 250 µm, whereas in the second one they are acanthose, from 200-280 µm, and no anatriaenes were found. A third species, *Tethyaclavigera*, with oscula (similar to porocalices) and no microxea was also described.
1922	Dendy	Description of *Cinachyravaccinata* and *C.providentiae* from the Indian Ocean. Both of them with microxea (no mention whether acanthose or not), being 200 × 4 µm in the former, and 220 × 5.5 µm in the latter one. *C.vaccinata* characterized by small hair-like protri- and prodiaenes, terminating in an elongated oval swelling tip unique to this species. *C.providentiae* with bottle-shaped porocalices.
1925	Wilson	Establishment of *Cinachyrella* as a subgenus of *Tetilla*, with type species *Tetillahirsuta* Dendy, 1889. The characters used to distinguish *Cinachyrella* species from the other were special depressions (=porocalices) and no specialization of a cortical zone. Wilson included the following species within *Cinachyrella: Cinachyramalaccensis* Sollas, 1902; *Tetillalimicola* Dendy, 1905; *Tetillaanomala* Dendy, 1905; *Cinachyraisis* Lendenfeld, 1907; *C.hamata* Lendenfeld, 1907; *C.alba-tridens* Lendenfeld, 1907; *C.alba-bidens* Lendenfeld, 1907; *C.alba-obtusa* Lendenfeld, 1907; *C.vertex* Lendenfeld, 1907; *Tetillacinachyroides* Hentschel, 1911; *Cinachyraphacoides* Hentschel, 1911; *Tethyaclavigera* Hentschel, 1912; *Cinachyramertoni* Hentschel, 1912; *Cinachyranuda* Hentschel, 1912; *Cinachyravaccinata* Dendy, 1922; *Cinachyraprovidentiae* Dendy, 1922. In addition, *Cinachyrellacrustata* and *Cinachyrellapaterifera* were described from Philippines. *C.crustata* with distinctive long and stout promonoenes, no microxea. *C.paterifera* with a characteristic cloaca (= large osculum) on top and root-like structure to attach to sediments, microxea (250 × 2 µm) observed in two specimens although almost absent in the third one of the type series, pointing out a high variability in the presence of microxea within the same individual.
1934	Burton	Taxonomic revision of *Cinachyraaustraliensis*. In his compilation, Burton grouped 16 nominal species described in 32 references and designated them as junior synonyms of the widespread species *C.australiensis*. Three different groups were recognized: the *australiensis*-group characterized by the presence of acanthose microxea; the *schulzei*-group with smooth microxea; and the *porosa*-group without microxea. Description of genus *Raphidotethya*.
1954	de Laubenfels	Identification of *Cinachyraporosa* and *Cinachyraaustraliensis* from Micronesia (West-Central Pacific).
1973	Thomas	Records of *Cinachyracavernosa* (Lamarck, 1815) from the Seychelles Islands, having, microxea (126 × 2 µm) sometimes granulated (= acanthose). Among the junior synonyms of *C.cavernosa*, Thomas included *Tethyacranium var. australiensis* Carter, 1886, *Chrotellaaustraliensis* Burton, 1937, and *Chrotellacavernosa* Burton, 1959. However, in the WPD (van Soest et al. 2018) *C.cavernosa* is still a valid species.
1982	Pulitzer-Finali	Description of *Cinachyratenuiviolacea* from the Great Barrier Reef (Australia), characterized by a light violet color, small oxeas (up to 2500 µm × 13-25 µm), atrophic anatriaenes, no microxeas, and no protriaenes in the choanosome.
1987	Rützler	Review of Family Tetillidae, including seven genera (all except for *Fangophilina*). SubgenusCinachyrella was elevated to the hierarchy of genus.
1992	Rützler and Smith	Review of four species of *Cinachyrella* for the Caribbean region, mainly described by Uliczka (1929). Geometry and size ranges of all spicule types were shown. According to their descriptions, *Cinachyrellakuekenthali* is the most similar species to *C.australiensis*, since both of them have acanthose microxea.
1994	Hooper and Wiedenmayer	Compilation of *Cinachyraaustraliensis* synonyms based on Burton (1934) taxonomic decision.
2002	van Soest and Rützler	Review of the eight genera of tetillids, including *Cinachyrella*. *Cinachyraaustraliensis* was transferred into the genus *Cinachyrella*. The authors considered that all junior synonyms proposed for *C.australiensis* by Burton (1934) should need further taxonomic revision. Moreover, the genera [*Psetalia*] Gray, 1873 (*nomem oblitum*), [*Labaria*] Gray, 1873 (nomen oblitum) and *Raphidotethya* Burton, 1934 were included as synonyms of the genus *Cinachyrella*.
2018	van Soest et al. (WPD)	Accepted synonyms of *Cinachyrellaaustraliensis* (Carter, 1886): *Tethyaaustraliensis* Carter, 1886; *Spirettaporosa* Lendenfeld, 1888; *Cinachyramalaccensis* Sollas, 1902; *Tetillalindgreni* Lendenfeld, 1903; *Tethyaarmata* Baer, 1906; *Cinachyraisis* Lendenfeld, 1907; *Tetillacinachyroides* Hentschel, 1911; and *Cinachyraprovidentiae* Dendy, 1922. Valid *Cinachyrella* spp. from the Indo-pacific (excluding species only found in the Red Sea) comprise 6 species
2018	This study	From our detailed examination of Indonesian material and type material, we conclude that in Indonesia there are three species: *Cinachyrellaaustraliensis* (Carter, 1886), *Cinachyrellaporosa* (Lendenfeld, 1888), and *Cinachyrellapaterifera* Wilson, 1922. Further investigations will reveal if the five species from the *C.schulzei*- group can be synonymized or belong to separate and distinctive species.

*Cinachyrellaaustraliensis* has been recorded from a wide geographic area from the Gulf of Oman (van Soest and Beglinger 2008), Thailand (Kritsanapuntu et al. 2001a-b, Putchakarn 2007), Vietnam (Azzini et al. 2007), Singapore (Lim et al. 2008), North Australia (McDonald et al. 2002), the Great Barrier Reef in Australia (Burton 1934), Southeast Australia (Carter, 1886), and Indonesia (e.g. Becking et al. 2006, de Voogd and Cleary 2008, de Voogd et al. 2009, Becking et al. 2013), inhabiting coastal mangroves, reefs, and marine lakes.

Ecological studies on the morphological plasticity of *C.australiensis* from North Australia (McDonald et al. 2002) and Thailand (Kritsanapuntu et al. 2001) have concluded that this species can adapt to extreme sedimentation and water current regimes through the variation of the body shape and reinforcement of spicules. Although these surveys showed interesting data on the individual sizes, porocalices, silica/organic content, both of them lack robust taxonomic data (type of spicules and their dimensions). It is therefore unclear whether the observed plasticity can be attributed to natural variation within the same species or may possibly be explained by different species inhabiting different habitats.

####### Cinachyrella australiensis

Taxon classificationAnimaliaTetractinellidaTetillidae

(Carter, 1886)

Figs 6 , 7

Tethya cranium var. australiensis Carter, 1886: 127 (holotype seen).
Tetilla ? australiensis ; Sollas, 1888: 43.
Spiretta raphidiophora Lendenfeld, 1888: 43 (type seen).
Tetilla hirsuta Dendy, 1889: 75 (type seen).
Tetilla ternatensis Lindgren, 1898: 329 pl. 17, fig. 14; pl. 19, Fig. 25 a-e, a’, b’. Ternate Not Tetillaternatensis; Kieschnick*, 1896: 527.
Tetilla australiensis ; Thiele, 1899: 6, pl.1 fig.1; pl. 5, fig.1 a-e. Celebes Sea.
Tetilla ternatensis ; Kirkpatrick, 1900: 132 (material seen) Not Tetillaternatensis Kieschnick*, 1896: 527.
Tetilla lindgreni Lendenfeld, 1903: 18.
Tetilla australiensis ; Lendenfeld, 1903: 20.
Tethya hebes Lendenfeld, 1907: 98, pl. XVI, figs 19–38. 19`South NW Australia, 91 m depth (syntype seen).
Cinachyra isis Lendenfeld, 1907: 143, pl. XV, figs 54–58, XVI, figs 1–4. Mermaid Strasse (NW Australia) (syntype seen); Dendy, 1922: 16, pl. 10, figs 3a-b.
Tetilla cinachyroides Hentschel, 1911: 281, textfig. 1. NW Australia, Barrow Island.
Cinachyra nuda Hentschel, 1912:333, pl. XIII, fig.2; pl. XVIII fig. 13. Aru Island (type seen).
Cinachyra vaccinata Dendy, 1922: 14, pl. 1, fig. 4; pl. 11, figs 1a-l. Diego Garcia, Chagos Island (type seen).
Cinachyra providentiae Dendy, 1922: 18, pl.1, figs 5–5a; pl. 10, figs2a–f. Providence Island (type seen).
Tetilla (Cinachyrella) hirsuta ; Wilson, 1925: 365, pl. 39, fig.4.
Cinachyra australiensis ; Burton, 1934: 523. In part, not C.australiensis in porosa-group, nor C.australiensis in schulzei-group; de Laubenfels, 1954: 241, text-fig. 166.
Cinachyrella anatriaenilla Fernandez, Kelly, Bell, 2017: 83, figs 2–4.

######## Material examined.

Holotype NHMUK 1886.12.15.367, Port Phillip Heads, Southeast Australia (as Tethyacraniumvar.australiensis). Holotype NHMUK 1886.8.27.634, Port Jackson, Sidney, Australia (as *Spirettaraphidiophora* Lendenfeld, 1888). NHMUK unreg. type, Gulf of Manaar, Sri Lanka (as *Tetillahirsuta* Dendy, 1889). NHMUK 1898.12.20.20 Christmas islands (as *Tetillaternatensis* Kirkpatrick, 1900). Holotype NHMUK 1908.9.24.19–21, 19°17'S 116°E, Gazelle Exp., Western Australia, (as *Tethyahebes* Lendenfeld, 1907). Syntype NHMUK 1908.9.24.74, Mermaid Strait, NW Australia (as *Cinachyraisis* Lendenfeld, 1907). RMNH unreg. fragment taken from the type (pers. comm. NJ de Voogd) and available in Naturalis collections, Aru Island, Indonesia, as *Cinachyranuda* Hentschel, 1912. Holotype NHMUK 1921.11.7.6, Diego Garcia, Chagos Islands (as *Cinachyravaccinata* Dendy, 1922). Holotype NHMUK 1921.11.7.8, Providence Island, Seychelles (as *Cinachyraprovidentiae* Dendy, 1922). **INDONESIA**. East Kalimantan, *Berau reef*, RMNH.POR.11101, RMNH.POR.11102, RMNH.POR.11103, RMNH.POR.11104, RMNH.POR.11105, RMNH.POR.11106, RMNH.POR.11107, RMNH.POR.11108, RMNH.POR.11109, RMNH.POR.11110, RMNH.POR.11111, RMNH.POR11112, RMNH.POR.11113, RMNH.POR.11114, RMNH.POR.11115, RMNH.POR.11116, RMNH.POR.11117, RMNH.POR.11210, RMNH.POR.11124, RMNH.POR.11125, RMNH.POR.11126, RMNH.POR.11127, RMNH.POR.11128, RMNH.POR.11129, RMNH.POR.11130, RMNH.POR.11118, RMNH.POR.11119, RMNH.POR.11120, RMNH.POR.11121, RMNH.POR.11122, RMNH.POR.11123; RMNH.POR.11132; RMNH.POR.11133, RMNH.POR.11134, RMNH.POR.11135, RMNH.POR.11136; *Pea Bay*, RMNH.POR.11162; *Haji Buang Lake*, RMNH.POR.11137, RMNH.POR.3511, RMNH.POR.3512, RMNH.POR.3513, RMNH.POR.3516, RMNH.POR.3517; *Kakaban Lake*, RMNH.*POR.*11161, RMNH.POR.11138, RMNH.POR.11139, RMNH.POR.11140, RMNH.POR.11141, RMNH.POR.11142, RMNH.POR.11143, RMNH.POR.11144, RMNH.POR.11145, RMNH.POR.11146, RMNH.POR.11147, RMNH.POR.11148, RMNH.POR.11149, RMNH.POR.11150, RMNH.POR.11151, RMNH.POR.11152, RMNH.POR.11153, RMNH.POR.11154, RMNH.POR.11155, RMNH.POR.11156, RMNH.POR.11157, RMNH.POR.11158, RMNH.POR.11159, RMNH.POR.11160. Java, *Thousand Islands*, RMNH.POR.1969. Ternate, *Ternate reef*, RMNH.POR.11308. Sulawesi, *Bunaken*, RMNH.POR.3108, RMNH.POR.3112, RMNH.POR.3119, RMNH.POR.3122. West Papua, *Sawaundarek Lake*, RMNH.POR.11163, RMNH.POR.11164, RMNH.POR.11165, RMNH.POR.11166, RMNH.POR.11167; *Gam Island, Wallace Lake*, RMNH.POR.11168, RMNH.POR.11169 *Outside Wallace Lake*, RMNH.POR.11170, RMNH.POR.11171, RMNH.POR.11172, RMNH.POR.11173; *Gam Island, Blue Water Mangrove*, RMNH.POR.11174, RMNH.POR.11175, RMNH.POR.11176, RMNH.POR.11177, RMNH.POR.11178, RMNH.POR.11179, RMNH.POR.11180, RMNH.POR.11181, RMNH.POR.11182, RMNH.POR.11183, RMNH.POR.11184, RMNH.POR.11185, RMNH.POR.11186, RMNH.POR.11187, RMNH.POR.11188, RMNH.POR.11189, RMNH.POR.11190, RMNH.POR.11191, RMNH.POR.11192; *Ctenophore Lake*, RMNH.POR.11193, RMNH.POR.11194, RMNH.POR.11195, RMNH.POR.11196, RMNH.POR.11197; *Outside Ctenophore Lake*, RMNH.POR.11198, RMNH.POR.11199, RMNH.POR.11200, RMNH.POR.11201; *Big Caulerpa Lake*, RMNH.POR.11202, RMNH.POR.11203; *Outside Big Caulerpa lake*, RMNH.POR.11204; *Gam Island*, RMNH.POR.11205, RMNH.POR.11206.

**Other material**: Singapore, RMNH.POR.3520, RMNH.POR.2440, RMNH.POR. 2505.

**Other types and material examined (not included as synonyms of *C.australiensis*)**: NHMUK 1892.8.8.8. Macclesfield Bank, South China Sea *Cinachyraschulzei* (unpublished material). Holotype NHMUK 1908.9.24.75 Red Sea, *Cinachyratrochiformis* Keller, 1891. Holotype NHMUK 1907.2.1.14, Gulf of Manaar, Sri Lanka, *Tetillapoculifera* Dendy, 1905. Holotype NHMUK 1912.2.1.35, Tella Tella Kebira, Red Sea, *Chrotellaibis* Row, 1911. RMNH unreg. fragment taken from the type (pers. comm. NJ de Voogd) available in Naturalis collections, Kei Island, Indonesia, *Cinachyramertoni* Hentschel, 1912.

######## Description.

**Figure 6.**
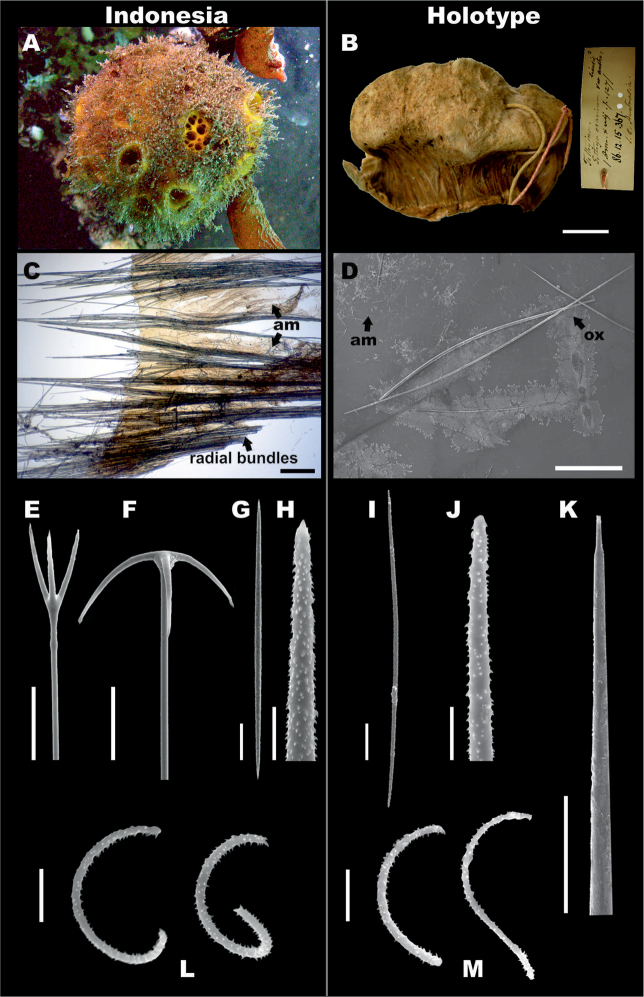
*Cinachyrellaaustraliensis*. **A, C, E-H, L**RMNH.POR.11139, Kakaban lake, Indonesia (left side) **B, D, I-K, M** holotype NHMUK 1886.12.15.367, Port Phillip Heads, Australia (right side) **A** In situ photograph showing porocalices **B** dry specimen, lateral view **C** skeleton showing acanthose microxeas (*am*) and radial bundles with oxeas **D** spicule montage showing acanthose microxeas (*am*), and oxeas (*ox*) **E** protriaene **F** anatriaene **G** Acanthose microxea, full lenght **H** acanthose microxea, detail **I** acanthose microxea, full length **J** acanthose microxea, detail **K** oxea, end detail **L, M** sigmaspires. Scale bars: 1 cm(**B**); 500 μm (**C, D**); 20 μm (**E–G, I**); 5 μm (**H, J, L, M**); 50 μm (**K**).

**External morphology.** Globular sponges, size from 4 to 10 cm in diameter (Figure 6A, B). Surface hispid due to the projecting spicules; covered by numerous porocalices. Porocalices are abundant bowl-shape with open oval apertures, up to 10 × 5 mm and 5 mm deep, or bottle-shape, up to 18 × 6.5 mm, with minuscule apertures (2–3 mm diameter), size of porocalices can vary between habitats; a cloaca, defined as a central exhalant cavity (Boury-Esnault and Rützler, 1997), is distinguishable at the top of some specimens (Figure 6A); in preserved material some porocalices are open. Color generally bright yellow when alive, which turns paler or even white in ethanol. In the field, the sponge can appear brownish due to sediment or greenish due to association with algae.

**Skeleton.** No cortex. Skeleton composed by bundles of oxeas and triaenes radiating from a central core.

**Megascleres.** Holotype and Indonesian specimens’ measurements are shown in Table 6. Holotype, oxeas 3375–4135.5–5500 mm × 15–24.7–37.5 mm (Figure 6D, K); no triaenes were observed in the type specimen; in Carter’s description, protriaenes are described (135 mm long) and the absence of anatriaenes was explained as their heads broke off when collected; Indonesian specimens have a wide size range of oxea 1000–5500 mm (Figure 6C), abundant anatriaenes (Figure 6F), with rhabd 2250–3224.4–4250 mm × 2.5–5.7–10 mm, cladi thin, mainly with obtuse angles 30–70.6–100 mm × 20–51.7–80 mm × 2.5–4.9–10 mm; protriaenes (Figure 6E), with thin and long cladi (20–57.1–80 mm × 25–86.9–170 mm × 2.5–7.5–12.5 mm), rhabd up to 5800 × 20 mm, tapering to dimensions of < 1 mm; few prodiaenes also observed, having smaller cladi (20–30 mm × 20–30 mm); no calthrop-like triaenes.

**Table 6.** Spicule measurements of eight specimens *Cinachyrellaaustraliensis*, five specimens of *C.anomala*, four specimens of *C.paterifera* from different regions (n = 10 per spicule type and dimension with minimum-mean-maximum). Asterisk (*) indicate that rhabd of spicules were broken and no measurement was possible. Double asterisk (**) indicate that a particular spicule type was not observed.

		*** Cinachyrella australiensis ***								*** Cinachyrella porosa ***					*** Cinachyrella paterifera ***			
		**BMNH86.12.15.367 (Holotype)**	**RMNH.POR.11120**	**RMNH.POR.11146**	**RMNH.POR.11123**	**RMNH.POR.11139**	**RMNH.POR.11118**	**RMNH.POR.11308**	**RMNH.POR.11192**	**BMNH86.8.27.632-3 (Holotype)**	**RMNH.POR.11226**	**RMNH.POR.11244**	**RMNH.POR.11262**	**RMNH.POR.11309**	**USNM21314 (Holotype)**	**RMNH.POR.11207**	**RMNH.POR.11213**	**RMNH.POR.11208**
**Locality**		Port Phillip Heads	Berau	Kakaban	Berau	Kakaban	Berau	Ternate	Raja Ampat	Port Denison	Tanah Banban	Teluk Pea	Raja Ampat	Ternate	Philippines	Berau	Raja Ampat	Berau
**Habitat**		Reef?	Reef	Marine Lake	Reef	Marine Lake	Reef	Reef	Mangrove	Reef	Marine Lake	Reef	Reef	Reef	Reef	Reef	Reef	Reef
**Oxeas**	Length	3375-**4135.5**-5500	1000-**3332**-4500	1375-**2912**-4000	2425-**3822.8**-5500	2250-**3066**-4250	2300-**4315**-5750	1500-**2676**-3800	2000-**2658.3**-3750	820-**2553.2**-3750	550-**2138.1**-3750	1350-**2702.5**-4000	1250-**2304.2**-3150	1500-**2710**-3500	1400-**3011.5**-4750	2850-**3580.6**-4500	1850-**3060**-5000	800-**2748.2**-4500
	Width	15-**24.7**-37.5	10-**40**-52.5	17.5-**33.9**-55	25-**41.7**-60	17.5-**26.4**-35	37.5-**59.1**-77.5	12.5-**30.3**-60	25-**31.3**-37.5	7.5-**29.4**-47.5	5-**28.2**-60	7.5-**29.5**-47.5	12.5-**29.4**-40	7.5-**32.6**-45	10-**34.5**-62.5	30-**46.1**-62.5	12.5-**35.6**-55	5-**34.2**-75
**Anatriaene**	Rhabd	**	2750-**3271.9**-3650	2250-**3317.9**-4250	2700-**3083.3**-3300	*	*	*	*	*	*	*	*	*	*	*	*	4250-**4250**-4250
	Rhabd width		5-**6**-7.5	2.5-**5.3**-7.5	4-**4.9**-5	5-**5**-5	5-**5**-5	7.5-**8.4**-10	5-**5.3**-7.5	2.5-**5.8**-7.5	10-**12**-15	5-**7.8**-10	5-**5**-5	5-**6.1**-7.5	5-**7.5**-10	5-**6.6**-10	7.5-**8.8**-10	5-**5**-5
	Cladi total		50-**63.5**-85	30-**74.2**-100	45-**58**-70	40-**55**-70	60-**81**-100	55-**79.4**-100	70-**83**-100	50-**67.6**-100	65-**71**-80	60-**65**-70	50-**62.9**-70	50-**62.5**-75	17.5-**24.2**-30	17.5-**37.5**-75	70-**90**-110	65-**79.3**-110
	Cladi length		40-**49.5**-65	20-**60.4**-80	30-**42.5**-50	35-**45**-60	32.5-**49.5**-57.5	30-**49.1**-67.5	50-**66**-80	30-**42**-60	45-**56**-65	40-**52**-70	40-**51.4**-60	30-**50.4**-62.5	2-**6.5**-10	7.5-**25.7**-80	50-**65**-80	42.5-**58.2**-90
	Cladi width		2.5-**4.5**-5	2.5-**3.5**-5	4-**5.6**-10	5-**5**-5	5-**5.1**-6	5-**5.6-**7.5	5-**5**-5	2.5-**5.6**-7.5	10-**10.5**-12.5	2.5-**6.6**-7.5	2.5-**4.6**-5	5-**5.4**-7.5	5-**5.8**-7.5	5-**6.4**-10	7.5-**7.5**-7.5	2.5-**4.3**-5
**Protriaene**	Rhabd length	**	3900-**4550**-5800	3700-**4262.5**-4750	840-**3522.5**-5000	2250-**2375**-2500	**	*	*	*	*	*	*	*	*	3500-**4210**-5350	*	4300-**4689.3**-5100
	Rhabd width		10-**13.3**-20	12.5-**14.7**-15	10-**12.5**-15	2.5-**5.9**-7.5		7.5-**8.3**-10	2.5-**5**-7.5	5-**7.3**-12.5	5-**8.8**-15	2.5-**6.5**-10	2.5-**3.6**-5	2.5-**5**-7.5	10-**10**-10	5-**9.5**-12.5	5-**7.8**-10	10-**15.2**-17.5
	Cladi total		20-**46.9**-70	20-**58.8**-80	40-**60.8**-80	40-**55**-80		45-**50.4**-55	25-**70.5**-100	25-**44.4**-65	20-**51.25**-80	40-**59**-80	30-**44.3**-60	50-**53.8**-67.5	30-**32.5**-35	35-**53.9**-75	40-**70**-100	30-**37.3**-60
	Cladi length		35-**66.3**-110	25-**93.8**-170	75-**95.8**-130	50-**79.5**-110		40-**77.9**-120	30-**108-**150	35-**73**-110	30-**77.5**-125	60-**100**-160	40-**67.1**-100	50-**78.3**-137.5	22.5-**31.3**-40	20-**74.1**-130	30-**82.5**-140	40-**51.6**-80
	Cladi width		5-**7.9**-10	10-**11.6**-12.5	10-**10.8**-12.5	2.5-**4.8**-5		5-**5.8**-7.5	2.5-**4**.3-5	5-**5.1**-7.5	5-**7.5**-15	2.5-**5.5**-10	2.5-**2.5**-2.5	2.5-**4**-5	7.5-**7.5**-7.5	2.5-**6.8**-7.5	5-**6.6**-7.5	10-**11.6**-15
**Strongyle**	Length	**	--	4000-**4000**-4000	2450-**3041.7**-4200			2200-**2650**-2850		2650-**2650**-2650	**	3350-**3350**-3350	**	**	*	2450-**2800**-3250	2100-**2975**-3700	1800-**1862.5**-1925
	Width			50-**50**-50	35-**43.3**-60			32.5-**39.4**-50		35-**35**-35		45-**45**-45			45-**45**-45	40-**43.1**-50	35-**45.3**-62.5	35-**37.5**-40
**Acanthose microxea**		117-**166.9**-260	160-**197.3**-230	150-**165**-200	200-**230**-270	150-**183.5**-240	157.5-**189.5**-225	170-**191.4**-225	137.5-**154**-175									
**Sigmaspires**		10-**14.4**-17.5	12.5-**15**-17.5	10-**12.3**-15	12.5-**14.8**-17.5	10-**12**-15	15-**16.5**-20	12.5-**15.7**-20	12.5-**14.8**-17.5	5-**8.6**-12.5	7.5-**8.5**-10	5-**8.9**-12.5	5-**8**-10	5-**8**-10	10-**13.2**-17.5	12.5-**15.3**-17.5	12.5-**14.5**-17.5	12.5-**16.2**-20
**Protriaene (hair-like)**	**Rhabd length**														*	*	*	550-**698.9**-820
	**Width**														2.5-**2.5**-2.5	2.5-**2.5**-2.5	2.5-**2.5**-2.5	2-**2.2**-2.5
	**Cladi total**														7.5-**12.5**-17.5	7.5-**10.9**-17.5	12.5-**13.6**-15	7.5-**11.7**-20
	**Cladi length**														12-**15.5**-20	17.5-**21.6**-25	12-**14.5**-17.5	10-**14.4**-25
	**Cladi width**														2.5-**2.5**-2.5	2-**2.1**-2.5	2.5-**2.5**-2.5	1-**1.7**-2.5

**Microscleres.** Numerous acanthose microxeas, holotype, 117–166.9–260 mm (Figure 6I, J), slightly larger in the Indonesian material 137.5–184.7–270 mm (Figure 6G, H); sigmaspires vary within the same range in both, holotype and Indonesian specimens, 10–14.4–20 mm, C-S shape (Figure 6L, M).

######## Ecology.

*Cinachyrellaaustraliensis* occurs in reefs, mangroves, and marine lakes, ranging in depths from 0 to at least 30 m, possibly deeper. Specimens can be covered by sand and mud; or in symbiosis with algae, resulting in green external color. This species produces 1–2 mm sized buds (Figure 8) and buds are extensively observed in specimens collected from marine lake habitats.

######## Distribution.

*Cinachyrellaaustraliensis* has a wide distribution in Indonesia, including Berau, Bunaken, Raja Ampat, Ternate, and Java. Previous Indonesian records are from Spermonde Archipelago in Sulawesi (de Voogd and Cleary 2005, Becking et al. 2006, de Voogd et al. 2006), North Sulawesi (Calcinai et al. 2017), Berau (de Voogd et al. 2009, Becking et al. 2013), Thousand Islands in Java (de Voogd and Cleary 2008), and Raja Ampat (Becking 2008). In addition, this species has also been found in Gulf of Oman (van Soest and Beglinger 2008), Seychelles Islands (Thomas 1973) Southwest Madagascar (Vacelet et al. 1976), Zanzibar (Pulitzer-Finali 1993), Thailand (Kritsanapuntu et al. 2001a-b, Putchakarn 2007), Singapore (Lim et al. 2008), Vietnam (Azzini et al. 2007), Philippines (Longakit et al. 2005), Northern Territory of Australia (McDonald et al. 2002), and the Great Barrier Reef in Australia (Burton 1934).

**Figure 7.**
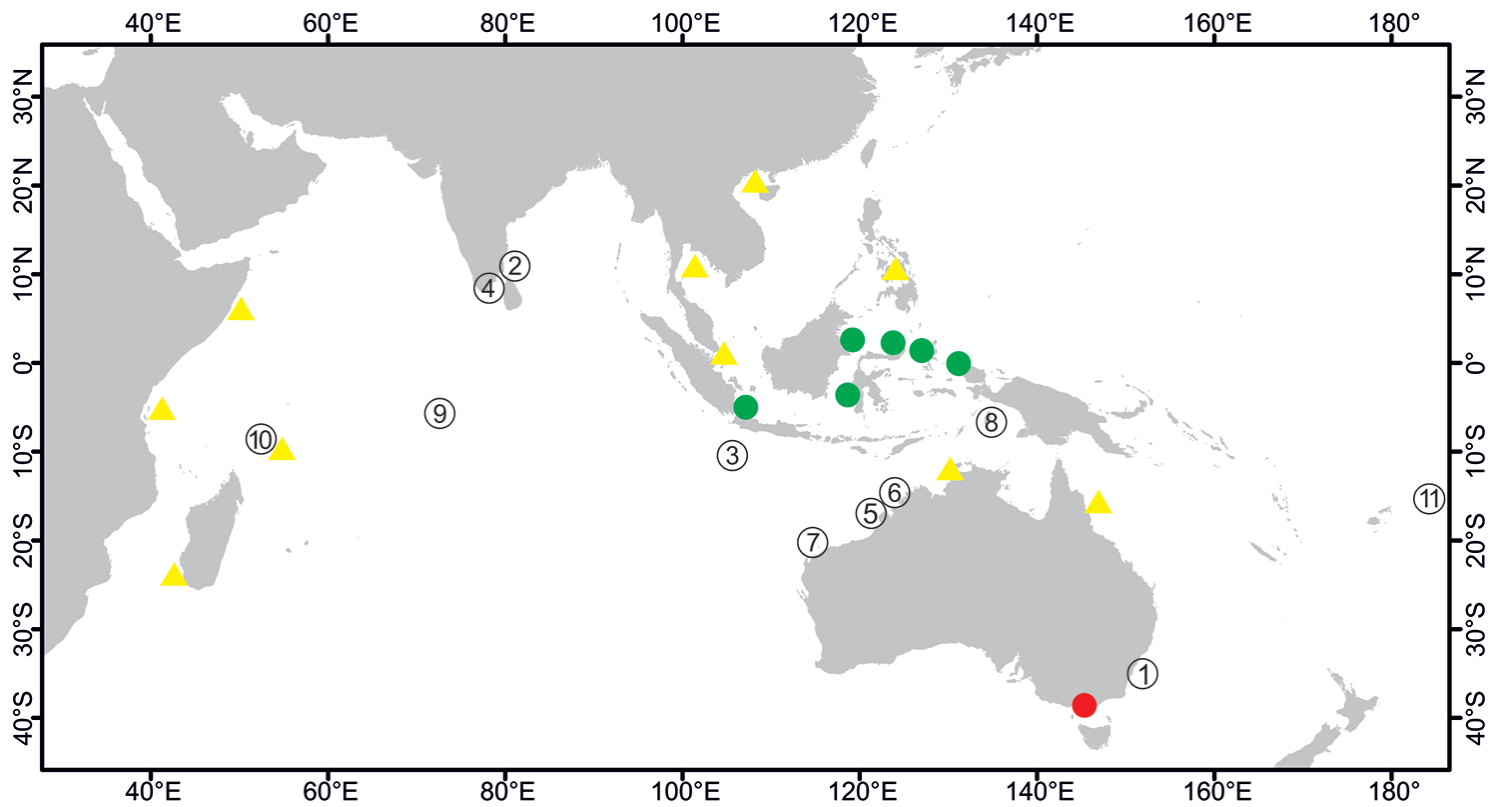
Distribution of *Cinachyrellaaustraliensis*. Red dot: type locality, Tethyacraniumvar.australiensis Carter, 1886, Port Phillip Heads, Southeast Australia. Green dots: Indonesian localities where the species was collected recently. Yellow triangles: Non-Indonesian localities, Seychelles Islands, Southwest Madagascar, Zanzibar, Thailand, Singapore, Vietnam, Philippines, Northern Territory of Australia, and the Great Barrier Reef in Australia. Circled numbers: type localities of synonymized species **1***Spirettaraphidiophora* Lendenfeld, 1888, Port Jackson, Sidney, Australia **2***Tetillahirsuta* Dendy, 1889, Gulf of Manaar, Sri Lanka **3***Tetillalindgreni* Lendenfeld, 1903, Christmas Island **4***Tetillapoculifera* Dendy, 1905, Gulf of Manaar, Sri Lanka **5***Tethyahebes*, 1907, at 19° South on the NW coast of Australia **6***Cinachyraisis* Lendenfeld, 1907, Mermaid Strait, NW Australia **7***Tetillacinachyroides* Hentschel, 1911, Barrow Island, NW Australia **8***Cinachyranuda* Hentschel, 1912, Aru Island, Indonesia **9***Cinachyravaccinata* Dendy, 1922, Diego Garcia, Chagos Islands **10***Cinachyraprovidentiae* Dendy, 1922, Providence Island, Seychelles **11***Cinachyrellaanatriaenilla* Fernandez, Kelly, Bell, 2017, American Samoa.

**Figure 8.**
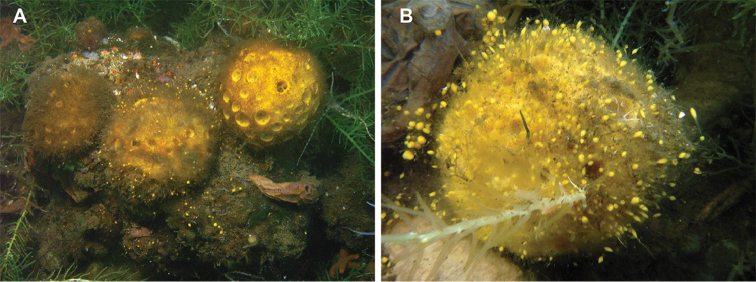
Budding and sediment capture of *Cinachyrella* species **A** Three individuals of *C.porosa* in Haji Buang lake, East Kalimantan, Indonesia, showing distribution of buds beyond the individuals and sediment capture **B** Close up of *C.porosa* with detail of buds. Each individual is approximately 4 cm in diameter.

######## Remarks.

In the type description of *C.australiensis*Carter (1886), the author did not observe anatriaenes as it can be interpreted from his statement: “I saw no anchors (smaller tetractinellids with recurved arms); but as their heads when exposed are generally broken off (for they catch in everything that they touch), it does not follow that they do not form part of the spiculation, particularly as they are present in most of the other species that I have been described (sic)”. We examined the holotype kept at the Natural History Museum (NHMUK 1886.12.15.367) and found neither anatriaenes nor protriaenes. In addition, most of the oxeas were broken in the type specimen. Within all the examined material there is a high variability in the presence or absence of triaenes without a distinct geographic pattern. This variation may be related to where the sponge was cut, as it seems that triaenes are particularly abundant around the porocalices compared to other parts of the sponge. These fragile spicules are also easily broken off. We still assign our specimens to the species *C.australiensis* due to the characteristic presence of acanthose microscleres. It is furthermore one of the most common names used in the literature since its description and without further evidence we do not want to cause more confusion. Further examination of *Cinachyrella* specimens from Australia, in particular from the type locality of *C.australiensis*, will shed more light in this situation. It is quite possible that after a review of specimens from Southern Australia, it will be evident that the Indonesian specimens that we assign to *C.australiensis* should in fact be assigned to another species. In that case one of the junior synonyms should be used, e.g. *C.raphidiophora* or *C.hirsuta*.

Although our focus was on Indonesian species, it was unavoidable to attempt, for the first time after Burton’s review (1934), check the status of his large list of junior synonyms, because some of them were described or later found in Indonesian localities. We gathered as many type specimens as possible, most of them repositories of the NHMUK (London) and NMNH (Washington DC). The main criteria we used to suggest a species as junior synonym of *C.australiensis* were the presence of acanthose microxea and that the mega- and micro-scleres have the same size range of the species. Therefore, here we include as junior synonyms the following species from Burton’s list: *Spirettaraphidiophora* Lendenfeld, 1888; *Tetillahirsuta* Dendy, 1889; *Cinachyraisis* Lenfenfeld, 1907; *Tetillacinachyroides* Hentschel, 1911; *Cinachyranuda* Hentschel, 1912; *Cinachyravaccinata* Dendy, 1922; *Cinachyraprovidentiae* Dendy, 1922. They all fulfill the *C.australiensis* description.

###### Here we provide further remarks on the following species, in chronologic order:

*Tetillalindgreni* Lendenfeld, 1903 was described as a new species to separate it from *T.ternatensis* Kieschnick, 1896, as *T.ternatensis*is is a *Paratetilla* based on the presence of calthrop-like spicules. Lendenfeld noticed that both, Lindgren’s (1898) and subsequently Kirkpatrick’s (1900) material, lack such calthrop-like spicules, and instead, they have acanthose microxea similar to other *Tetilla* specimens described in his monograph (Lendenfeld 1903). From that material, we checked Kirkpatrick’s specimens and suggest that *T.lindgreni* is a junior synonym of *C.australiensis*.

*Tethyahebes* Lendenfeld, 1907 has acanthose microxea and it has most of *C.australiensis* characters, yet it was excluded from Lendenfeld’s Cinachyrinae-group (with porocalices) because he did not observe porocalices. The type specimens of *T.hebes* examined at the NHM (NHMUK 1908.9.24.66) are two small fragments, about 1.2 × 1 cm, and it is not possible to observe neither discard the presence of porocalices. Apart from that, the general skeletal arrangement and spicule configuration suggest that *T.hebes* fulfil all other morphological characteristics of *C.australiensis*. Therefore, we suggest that *T.hebes* is a junior synonym of *C.australiensis*.

We exclude from *C.australiensis* some junior synonyms that are part of the *schulzei*-group species proposed by Burton (1934). These species have smooth microxea and include Keller’s (1891) species from the Red Sea, *Cinachyraschulzei* and *Cinachyratrochiformis*. The taxonomic case of *C.schulzei* becomes more complicated as Kieschnick (1898, 1900) described a new species named *Tetillaschulzei* from material collected in Amboine islands of Indonesia with porocalices and spicules diagnostic of *Cinachyrella*, including microxea. However, Kieschnick did not mention any observation whether or not the microxea of *T.schulzei* have acanthose surface. The set of characters of *Cinachyraschulzei* Keller, 1891 and *Tetillaschulzei* Kieschnick, 1898 correspond to *Cinachyrella*. However, we consider that both species should be treated as homonyms because they were described under two different genera, from different and distant localities and we were not able to find their type material to verify if they could be synonymized. Other species within the *schulzei*-group are *Cinachyramertoni* Hentschel, 1912 from Kei island in Indonesia; *Tetillapoculifera* Dendy, 1905 from Sri Lanka; and *Chrotellaibis* Row, 1911 from the Red Sea. Special attention and a further revision is proposed for the *schulzei*-group of species, as we did not observe any specimen of the genus *Cinachyrella* with smooth microxea within the Indonesian material examined in this study. It is important to mention that thin smooth microxea were observed in both *Paratetilla* species, *P.bacca* and *P.arcifera*, but they also have calthrops as a diagnostic character of the genus.

We also exclude from *C.australiensis* two of the junior synonyms still present in the WPD (van Soest et al. 2018). First, *Tethyaarmata* Baer, 1906, because it is clear from the description that this species has a proteinous cortex reinforced by microxeas, resembling other *Craniella* species. Second, we exclude the junior synonym *Cinachyramalaccensis* Sollas, 1902, as the description does not mention the presence of microxea, therefore we suggest to synonymise it with *C.porosa*.

In our view, the recently erected species of *Cinachyrellaanatriaenilla* is junior synonym of *C.australiensis*, because the oxea and the microscleres fall within the size range of the type species of *C.australiensis* as well as the specimens we have included in this review. The authors distinguish their species from *C.australiensis* on the basis of having only one category of oxeas versus two categories in *C.anatriaenilla*. However, we do not recognize size classes in oxea in any of the *Cinachyrella* specimens and types, but rather a continuos range in size (1000–5500 mm for *C.australiensis*). The oxea of *C.anatriaenilla* fall within the size range of the type specimen of *C.australiensis* as well as the other reviewed material of *C.australiensis*. In addition, the authors based their statements on the revision of the type specimen of *C.kuekenthali*, which is from the west Atlantic, but they did not review the type specimen of *C.australiensis* nor any of the other species with acanthose microxea from the Indo-Pacific.

Recent molecular studies (Szitenberg et al. 2013, Schuster et al. 2017) show that *Cinachyrella* is a polyphyletic genus. It is beyond the scope of the current study to review the taxonomic status of the genus *Cinachyrella*. Within *C.australiensis* there are different genotypes (Schuster et al. 2017) that possibly represent morphologically cryptic species. Among the high morphological variation observed within our Indonesian specimens, some trends could be highlighted among the different populations. For instance, specimens from reefs of Berau were generally larger (up to 8 cm in diameter) and their porocalices had a bottle-shape with a small aperture (1 to 4 mm) and the cavity was often occupied by a shrimp. Although these characteristics resemble *C.providentiae*, the latter is one of the junior synonyms that we propose for *C.australiensis* based on spicule dimensions and forms. Specimens from Raja Ampat generally had smaller acanthose microxeas (Table 6), while in some specimens collected in marine lakes few abnormal spicules were observed. Yet, in all cases we could not detect consistent, quantifiable morphological differences.

####### Cinachyrella porosa

Taxon classificationAnimaliaTetractinellidaTetillidae

(Lendenfeld, 1888)

Figs 9 , 10

Spiretta porosa Lendenfeld, 1888: 43 (type seen).
Cinachyra malaccensis Sollas, 1902: 219, pl. XIV, fig. 2; pl. XV, fig. 5. Malacca Strait.
Tetilla porosa ; Lendenfeld, 1903: 22.
Tetilla anomala Dendy, 1905: 91, pl. III, fig.5 (type seen).
Cinachyra albatridens Lendenfeld, 1907: 149, pl. XV, figs 7–9 (type seen).
Cinachyra albaobtusa Lendenfeld, 1907: 154, pl. XVI, figs 45–52 (type seen).
Cinachyra albabidens Lendenfeld, 1907: 151, pl. XVI, figs 39–44 (type seen).
Tethya clavigera Hentschel, 1912: 327, pl. XVI, fig.1, pl. XVIII, fig. 10 In Aru Island, Beach Ngaiboor Trangan.
Cinachyra anomala ; Dendy, 1922: 20, pl. 1, fig. 3 (material seen).
Cinachyra porosa ; de Laubenfels, 1954: 240, pl. XI, fig. b (material seen).

######## Material examined.

Holotype NHMUK 1886.8.29.632-633, Port Denison, Australia (as *Spirettaporosa*). NHMUK 1907.2.1.12, Chilaw, Sri Lanka (as *Tetillaanomala*). NHMUK 1908.2.9.40-42, Diego Garcia, Chagos Archipelago (as *Cinachyraalbatridens*). NHMUK 1908.9.24.72, Anachoreten (=Keniet) Islands, Papua New Guinea (as *Cinachyraalbaobtusa*). NHMUK 1908.9.24.71, Tonga Islands (as *Cinachyraalbabidens*). **INDONESIA**, East Kalimantan, *Berau reef*, RMNH.POR.11228 [LT628324]; *Pea Bay*, RMNH.POR.11242, RMNH.POR.11243, RMNH.POR.11244 [JX177888]; *Bamban Lake*, RMNH.POR.11222, RMNH.POR.11223, RMNH.POR.11224, RMNH.POR.11225 [LT628327], RMNH.POR.11226; RMNH.POR.11226; *Bandong Lake*, RMNH.POR.11227; *Haji Buang Lake*, RMNH.POR.11236, RMNH.POR.11237, RMNH.POR.11238, RMNH.POR.11239, RMNH.POR.11240 [LT628325], RMNH.POR.11230, RMNH.POR.11231, RMNH.POR.11232 [LT628326], RMNH.POR.11233, RMNH.POR.11234, RMNH.POR.11235, RMNH.POR. 3514; *Kakaban Lake*, RMNH.POR.11241. Java, *Thousand Islands*, RMNH.POR.1998, RMNH.POR.2108. Sulawesi, *Bunaken*, RMNH.POR.3105. Ternate, *Ternate reef*, RMNH.POR.11309. West Papua, *Sawaundarek Lake*, RMNH.POR.11245 [JX177884], RMNH.POR.11246 [LT628323], RMNH.POR.11247, RMNH.POR.11248; *Ctenophore Lake*, RMNH.POR.11249, RMNH.POR.11250, RMNH.POR.11251, RMNH.POR.11251, RMNH.POR.11252, RMNH.POR.11253, RMNH.POR.11254, RMNH.POR.11255, RMNH.POR.11256, RMNH.POR.11257, RMNH.POR.11258, RMNH.POR.11259; *Outside Ctenophore Lake*, RMNH.POR.11260, RMNH.POR.11261, RMNH.POR.11262; *Gam Island, Reef flat*, RMNH.POR.11263; *Gam Island, Mangrove*, RMNH.POR.11264.

######## Description.

**Figure 9.**
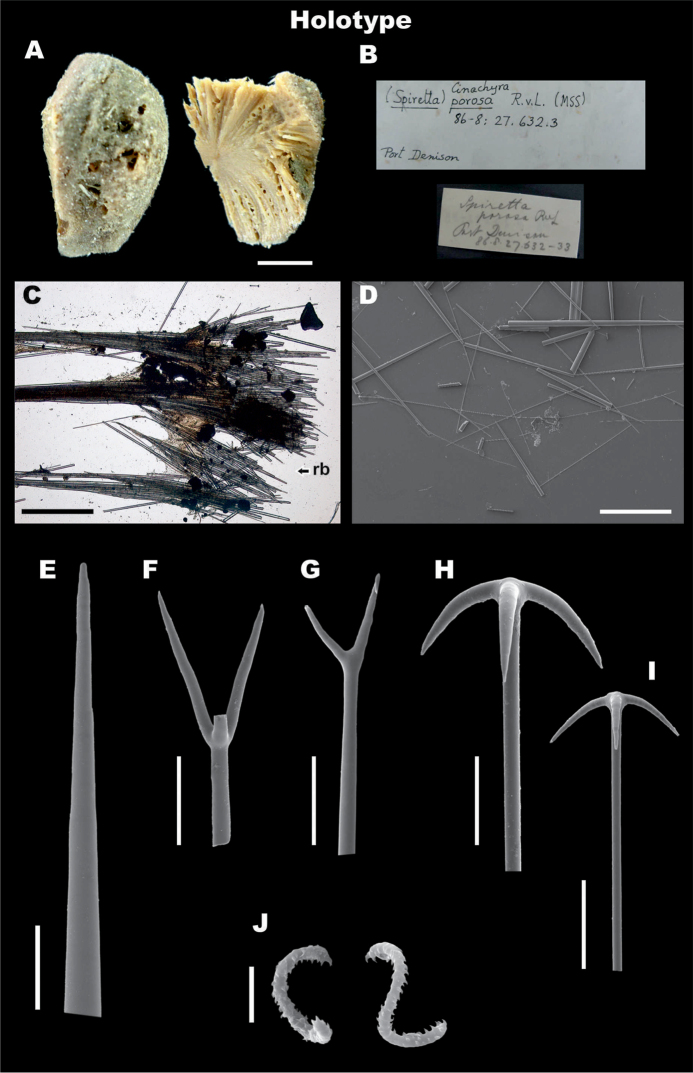
*Cinachyrellaporosa*. Holotype NHMUK1886.8.29.632-633, Port Denison, Australia. **A** preserved material showing porocalices and internal structure **B** Labels of the type specimen **C** skeleton **D** electron micrograph showing oxea fragments and triaenes rhabds **E** oxea, end detail **F** protriaene **G** prodiaene **H, I** anatriaenes **J** sigmaspires. Scale bars: 1 cm (**A, C**); 500 μm (**D**); 50 μm (**E**); 40 μm (**F–I**); 5 μm (**J**).

**Figure 10.**
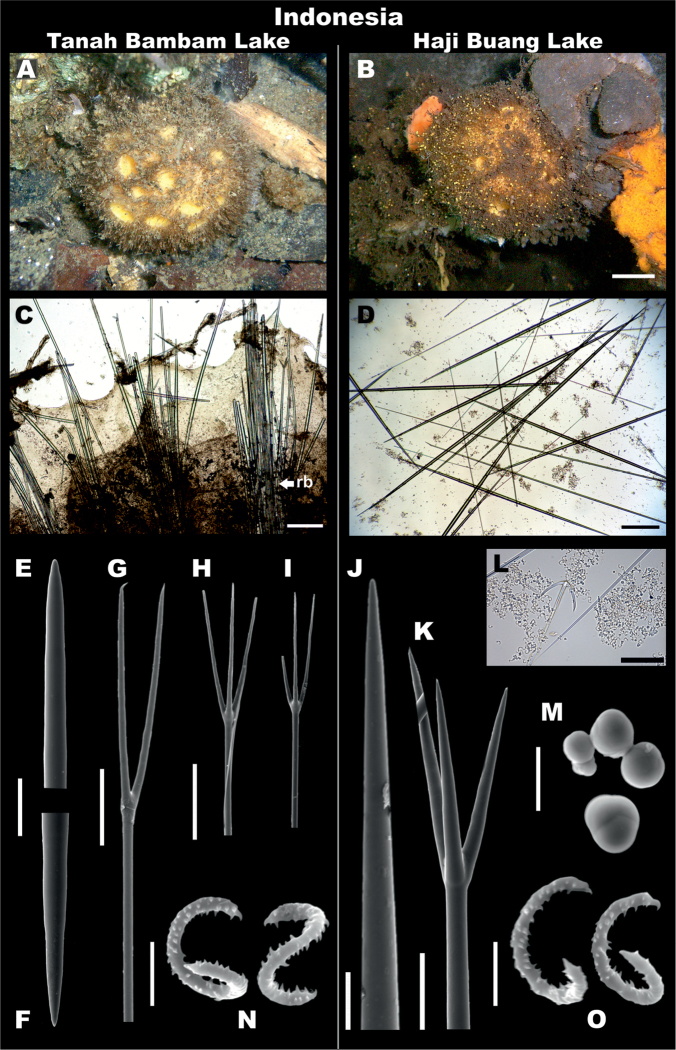
*Cinachyrellaporosa* from Indonesia. A, C, E-I, N, RMNH.POR.11223, Tanah Bambam Lake. **B,D, J-M, O**RMNH.POR.11235, Haji Buang Lake **A-B***In situ* photographs; **C** skeleton, showing radial bundles and triaenes **D** spicules in light microscope showing oxeas and triaenes rhabds **E, F** oxea, end details **G** prodiaene **H, I** protriaene **J** oxea, end detail **K** protriaene **L** anatriaene in light microscopy **M** spheres **N, O** sigmaspires. Scale bars: 500 μm (**C, D**); 20 μm (**E,F, J**); 40 μm (**G–I, K**); 100 μm (**L**); 5 μm (**M–O**).

**External morphology.** Globular sponges, size from 3 to 5 cm in diameter (Figs 9A, 10A, B). Surface highly hispid due to the projecting spicules, covered by numerous porocalices. Porocalices are bowl-shape, with rounded apertures, up to 4 × 5 mm and 5 mm deep, abundant; no cloaca; in preserved material some porocalices are closed. Color generally yellow when alive (Figure 10A, B), which turns paler or even white-grey after preservation in ethanol (Figure 9A).

**Skeleton.** No cortex. Skeleton composed by bundles of oxeas and triaenes radiating from a central core (Figs 9C, 10C).

**Megascleres**. Measurements are shown in Table 6 for the holotype and Indonesian specimens. Holotype, oxeas 820–2553.2–3750 mm × 7.5–29.4–47.5 mm (Figure 9C-E); few anatriaenes (Figure 9H, I), with rhabd always broken 2.5–7.3–15 mm, cladi thin, with obtuse angles 50–67.6–100 mm × 30–42–60 mm × 2.5–5.6–7.5 mm; protriaenes less abundant (Figure 9F), with rhabd always broken up to 5800 mm × 5–7.3–12.5, probably tapering to dimensions < 1 mm, with thin and long cladi (25–44.4–65mm × 35–73–110mm × 5–5.1–7.5 mm); abundant prodiaenes with similar dimensions as protriaenes (Figure 9G).

**Microscleres.** No microxeas. Sigmaspires 5–8.6–12.5 mm in the holotype (Figure 9J) and 5–8.4–12.5 in the Indonesian specimens (Figure 10N, O), C-S shape; in some Indonesian specimens, silica spheres ranging from 3–7 mm in diameter can be present (Figure 10M).

######## Ecology.

Occurs in reefs, mangroves, and marine lakes. Predominantly in shallow areas. Notably, a large population inhabit the marine lake of Tanah Bambam, where *C.porosa* was the dominant representative of moon sponges. This species produces 1–2 mm sized buds (Figure 8) and buds extensively in marine lakes habitats.

######## Distribution.

According to the material examined in this revision, we observed that this species is widely distributed in the Indo-Pacific, from the Chagos archipelago, Sri Lanka, Australia, and Tonga Islands. In Indonesia, *C.porosa* has been collected in East Kalimantan, Java, Ternate, and West Papua.

**Figure 11.**
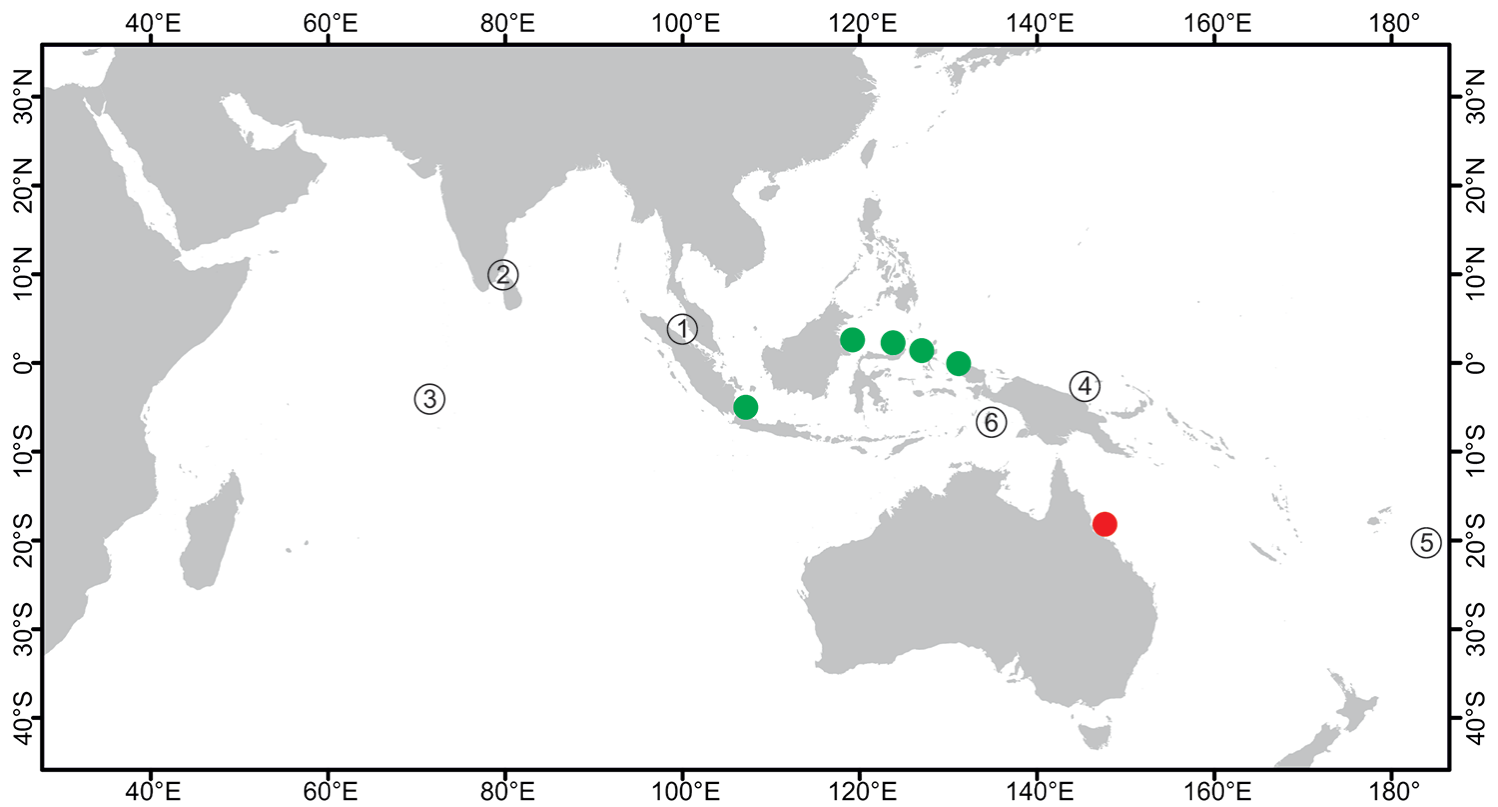
Distribution of *Cinachyrellaporosa*. Red dot: type locality, *Spirettaporosa* Lendenfeld, 1888, Port Denison, Queensland, Australia. Green dots: Indonesian localities where the species was collected recently. Circled numbers: type localities of synonymized species **1***Cinachyramalaccensis* Sollas, 1902, Malacca Strait, Malaysia **2***Tetillaanomala* Dendy, 1905, Chilaw, Sri Lanka **3***Cinachyraalbatridens* Lendenfeld, 1907, Diego Garcia, Chagos Archipelago **4***Cinachyraalbaobtusa* Lendenfeld, 1907, Anachoreten (=Keniet) Islands, Papua New Guinea **5***Cinachyraalbabidens* Lendenfeld, 1907, Tonga Islands **6***Tethyaclavigera* Hentschel, 1912, Aru Island, Indonesia.

######## Remarks.

**Figure 12.**
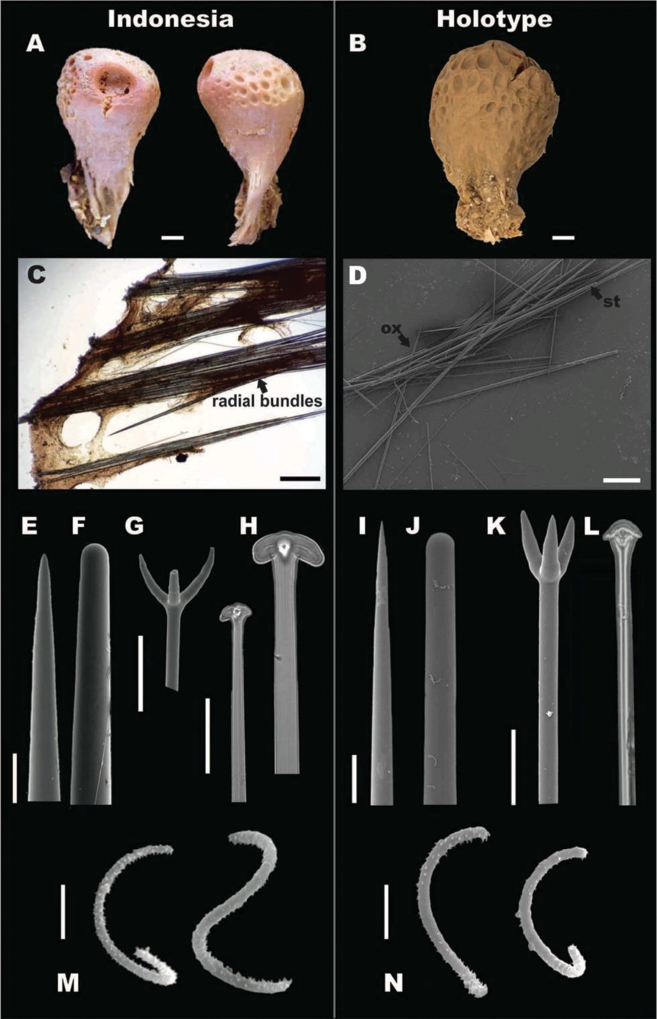
*Cinachyrellapaterifera*. **A, C, E-H, M**RMNH.POR.11207, Berau Reef, Indonesia (left side). **B, D, I-L, N** holotype USNM 21314, Timundao Reef, Sulu Archipelago, Philippines (right side) **A** specimen recently collected showing typical pink color, porocalices and stalk **B** Holotype, showing porocalices and stalk **C** skeleton showing radial bundles **D** spicules showing oxeas (ox) and strongyle (st), (scale bar 500 μm); **E** oxea, end detail **F** strongyle, end detail **G** protriaene **H** anatriaenes with short or abnormal cladus **I** oxea, end detail **J** strongyle, end detail **K** protriaenes **L** anatriaene with short or abnormal cladus **M, N** sigmaspires. Scale bars: 1 cm (**A, B**); 500 μm (**C, D**); 40 μm (**E–L**); 5 μm (**M, N**)

**Figure 13.**
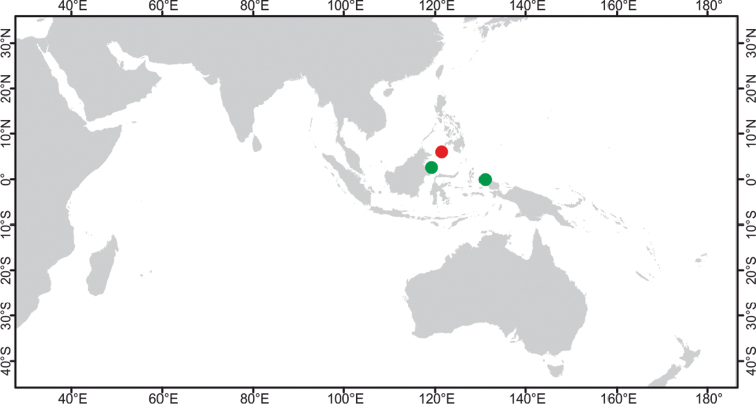
Distribution of *Cinachyrellapaterifera*. Red dot: type locality, Tetilla (Cinachyrella) paterifera Wilson, 1925, Sibutu Island, Philippines. Green dots: Indonesian localities where the species was collected recently.

*Cinachyrellaporosa* is distinguished from *C.australiensis* by the absence of acanthose microxea and smaller size of sigmaspires. The first species described with these two diagnostic characteristics was *Spirettaporosa* Lendenfeld, 1888, subsequently transferred to the genus *Tetilla* (Lendenfeld 1903) and included as a junior synonym of *C.australiensis* in both, Burton (1934) and WPD (2018). The detailed examination of the holotype of *C.porosa* suggests that this species should therefore be resurrected. Based on the careful examination of the holotypes of *C.albabidens* (Lendenfeld, 1907) and *C.albaobtusa* (Lendenfeld, 1907), and the descriptions and plates of *C.malaccensis* (Sollas, 1902) and *C.clavigera* (Hentschel, 1912), we coincide with the *porosa*-group recognized by Burton (1934). However, we disagree with the statement that intermediate forms can be found within the wide range of variation of *C.australiensis*, and therefore we consider *C.porosa* as a valid species clearly differentiated from *C.australiensis*. Lendenfeld (1907) recognized the difficulties to separate the three species of the alba-group, and his decision to discriminate them as different species was based on distant localities and slight differences on the abundance of triaenes. After the morphological analysis of the *C.albatridens* holotype, we consider that this species could also be a junior synonym of *C.porosa* because neither microxea nor other characters to separate this species were found. Although Burton (1934) did not consider *C.anomala* (Dendy, 1905) within the *porosa*-group, we suggest that a similar decision could be made based on our observations of the type specimen. Some of the Indonesian specimens have silica micro-spherules. Similar spherules have been described for species *C.anomala* and *C.hirsuta* (Dendy, 1905), as well as *Tetillacinachyroides* (Hentschel 1911). Because *C.hirsuta* and *T.cinachyroides* contain acanthose microxea, they are synonimized with *C.australiensis*. The nature of these spherules has been discussed by Dendy (1905) and Lendenfeld (1907). Dendy (1905) suggests that the spherules are associated with mother cells, which probably would give origin to sigmaspires, or they can be considered as anomalous or incidental spicules. On the other hand, Lendenfeld (1907) estimated that spherules are the earlier stages of oxeas as described for *Tethyacranium* (see Lendenfeld 1907, plate 14 figs 11–15). Silica spherules are very variable within populations of the same species and among different genera in Tetillidae, suggesting that this character has no taxonomic value.

####### Cinachyrella paterifera

Taxon classificationAnimaliaTetractinellidaTetillidae

(Wilson, 1925)

Figs 12 , 13

Tetilla (Cinachyrella) paterifera Wilson, 1925: 375; plate 39, figs 6, 8; plate 48, fig. 4 (type seen).

######## Material examined.

Holotype USNM21314, South of Tumindao Reef, Tibutu Island, Sibutu Group, Sulu Archipelago, Philippines, 18 m, 27 Feb 1908. **INDONESIA**. East Kalimantan, *Berau reef*, RMNH.POR.11207; RMNH.POR.11208; RMNH.POR.11209; RMNH.POR.11211. West Papua, *Wallace Lake*, RMNH.POR.11212, RMNH.POR.11213, RMNH.POR.11214; *Outside Wallace Lake*, RMNH.POR.11215; *Gam Island*, RMNH.POR.11216, RMNH.POR.11217, RMNH.POR.11218, RMNH.POR.11219, RMNH.POR.11220; *Ctenophore Lake*, RMNH.POR.11221.

######## Description.

**External morphology**. Globular sponges, size from 5 to 7 cm in diameter attached to the substrate by a large peduncle/shaft 3 × 2.5 cm (Figure 12 A, B). Surface smooth to hispid due to the projecting spicules, covered by porocalices. Porocalices are bowl or pocket-shape, with rounded apertures, up to 5 × 7 mm and 2–4 mm deep; a central cloaca is located on the top, 15 × 12 mm in diameter and 10 mm deep. Color bright pink when alive, which turns slightly paler in ethanol. Skeleton composed by bundles of oxeas and triaenes radiating from a central core. No cortex.

**Megascleres.** The holotype and Indonesian measurements are shown in Table 6. Holotype, oxeas 1400–3011.5–4750 mm × 10–34.5–62.5 mm (Figure 12D, I); few anatriaenes (Figure 12L), with a thick, small, poorly developed cladi, 17.5–24.2–30 mm × 2–6.5–10 mm × 5–5.8–7.5 mm, rhabd slightly thicker in the middle 15–25 mm, and tapering to dimensions of < 1 mm.; two different types of protriaenes, first one rare, with thick and small cladi (Figure 12K), 30–32.5–35 mm × 22.5–31.3–40 mm × 7.5–7.5–7.5 mm, rhabd usually broken, up to 5000 × 10 mm, thicker in the middle 40 mm, and tapering to dimensions of < 1 mm, the second type smaller, very abundant around porocalices, with small cladi in acute angle (fork-shape), 7.5–12.5–17.5 mm × 12–15.5–20 mm × 2.5–2.5–2.5 mm, rhabd up to 820 × 2.5 mm; strongyles are common, although only broken spicules observed in the holotype (Figure 12J), Indonesian specimens are 1800–2545.8–3700 mm × 35–42.7–62.5 mm (Figure 12F); no calthrop-like triaenes.

**Microscleres.** No microxeas; sigmaspires 10–13.2–17.5 mm in the holotype (Figure 12N) and 10–14.8–20 mm in Indonesian material (Figure 12M); C-S shape.

######## Ecology.

The species occurs mainly in reefs, and it is rare in marine lakes and mangroves. It usually inhabits sand bottoms, in which the penduncle serves as a support structure.

######## Distribution.

Indonesia, including East Kalimantan and West Papua. It is also known from Sibutu Island in Philippines (Wilson 1925). Although it is found in a variety of habitats, *C.paterifera* is the least common species of *Cinachyrella* from Indonesia.

######## Remarks.

*Cinachyrellapaterifera* has a characteristic elongated peduncle, it is pink to violet colored, and it contains abnormal anatriaenes. Interestingly, Wilson (1925) described rare microxeas (250 × 2 µm) in one specimen of the type series, whilst they were very abundant in the other two types. After a detailed examination of the type specimen USNM 21314 and preparations from different parts of the individual sponge, no microxeas were observed, suggesting that this character is not diagnostic of the species. Although *C.tenuiviolacea* (Pulitzer-Finali 1982) from the Great Barrier Reef resembles *C.paterifera* in the distinctive pink to violet color and presence of abnormal anatriaenes, it remains to be investigated if these two species could be synonymized. We could not access type material from *C.tenuiviolacea*, and from the bad conditions of preservation noted by Pulitzer-Finali (1982) in his type specimen, it is not possible to determine whether the specimen has or does not have the peduncle characteristic of *C.paterifera*. The large numbers of hair-like protri- and prodiaenes around the porocalices of *C.paterifera*, resemble those described for *C.vaccinata* (Dendy, 1905), yet the *C.vaccinata* type contains acanthose microxea characteristic of *C.australiensis*. *Cinachyrellapaterifera* share with *C.porosa* the absence of microxea, but they differ by the larger sigmaspires and abnormal protriaenes of *C.paterifera*. Indonesian specimens vary within the morphological range of the species. Specimens of this species belong to the same phylogenetic clade supporting its monophyly (Szitenberg et al. 2013; Schuster et al. 2017).

###### Identification key for Indonesian *Paratetilla* and *Cinachyrella* species

**Table:** 

1	Porocalices present; calthrops	**2**
–	Porocalices present; no calthrops, all triaenes –if present– are long-shafted	**4**
2	Trichodragmata present	*** Paratetilla corrugata ***
–	Trichodragmata not present	**3**
3	High numbers of porocalices, small size (up to 5 mm), brown color	*** Paratetilla bacca ***
–	Few porocalices, large size (7–15 mm), orange color, fleshy consistency	*** Paratetilla arcifera ***
4	Microxea present	**5**
–	Microxea not present	**6**
5	Acanthose microxea present (115–270 μm); sigmaspires 10–20 μm	*** Cinachyrella australiensis ***
–	Smooth microxea	***Cinachyrellaschulzei***-**group**
6	Small sigmaspires (5–10, few up to 12.5 μm), generally yellow color and ball-shape	*** Cinachyrella porosa ***
–	Large sigmaspires (10–20 μm), generally pink color, sometimes with peduncle to attach it to the substrate, pear-shape; protriaenes in two different classes; few anatriaenes with reduced and deformed cladi	*** Cinachyrella paterifera ***

###### Final remarks

Our results contribute to the understanding of the taxonomy and systematics of the Indo-Pacific tetillids. A review of the taxonomic history of the genus *Paratetilla* and the species *Cinachyrellaaustraliensis*, showed some cases of misinterpreted synonyms, misidentifications and lack of detailed descriptions for some species. The concept of a single widespread species is refuted for *Paratetillabacca* (Dendy 1922, Burton 1959) as well as for *Cinachyrellaaustraliensis* (Burton 1934). A wide morphological variation within moon sponges was observed for specimens collected in Indonesia. Among our material, we recognize three *Paratetilla* and four *Cinachyrella* species occurring in Indonesia, inhabiting a variety of habitats such as marine lakes, coral reefs, and mangroves. We are resurrecting *P.arcifera*Wilson 1925 and *C.porosa* (Lendenfeld, 1888) as valid species. The majority of the holotypes were studied for the current study; the ones we did not review were either unavailable or the description of the text was clear and comprehensive.

The species of *Paratetilla* and *Cinachyrella* are clearly highly adaptable and widely distributed sponges. All species in the current study are distributed across Indonesia. It is remarkable that they are all sympatric, some species occuring together in the same marine lake. We have reviewed specimens from East Kalimantan, North Sulawesi, and West Papua. It is highly likely that there are more species in Indonesia in regions that have not been sampled as extensively. Further investigations into *Paratetilla* and *Cinachyrella* from the Molluccas, Nusa Tenggara, South Kalimantan, Eastern Papua, and also the virtually unexplored deep sea of Indonesia, will likely lead to the discovery of more species within these genera. Most species occur in all studied habitats (marine lakes, mangroves, and reefs) with a high degree of tolerance for high temperature and sedimentation, as has been observed in other families of sponges (Schönberg 2015). The exceptions to this high tolerance were *P.arcifera* and *C.paterifera*, which were only seen in reefs with little sedimentation or sediment resuspension. High budding was observed in specimens of *Cinachyrellaaustraliensis* and *C.porosa* residing in marine lakes, while no budding was observed in the same species in the reefs. Singh and Thakur (2015) revealed temperature as the most prominent factor regulating the intensity of budding in Cinachyrellacf.cavernosa.

Previous molecular phylogenetic studies indicate that *P.bacca, P.arcifera, C.porosa*, and *C.paterifera* are distinct monophyletic species, while *Cinachyrellaaustraliensis* may consist of a species complex with morphologically cryptic species (Schuster et al. 2017). In the specimens that we identify as *C.australiensis* we do not find any consistent differences in spiculation to validate distinct species, in spite of the different haplotypes that are found within our specimens. Carella et al. (2016) also found that several well-supported subgroups within the *Cinachyrella* clade might correspond to subgenera. We were not able to distinguish multiple species with our set of *C.australiensis* specimens using standard morphological characters. Among the reviewed literature, we also observed that there is a tendency among people making inventories of reef species to name any yellow or yellow-orange tetillid ball *C.australiensis*. It is clear that the genus *Cinachyrella* and in particular the species *C.australiensis* require further analysis using either other molecular markers or morphological characters that go beyond the aims of the current study. We hope that our detailed study, images, and key will ensure that species from *Paratetilla and Cinachyrella* will be identified correctly based on morphological characters. It is important to understand the distinction between species, as there is a growing interest in natural products and other biobased studies from tetillids (e.g. Cleary et al. 2013, Mokhlesi et al. 2017, Zhang et al. 2017). We expect that the current study can provide a solid basis for subsequent species descriptions of Indo-Pacific species of the genera *Cinachyrella* and *Paratetilla*.

## Supplementary Material

XML Treatment for Paratetilla bacca

XML Treatment for Paratetilla arcifera

XML Treatment for Cinachyrella australiensis

XML Treatment for Cinachyrella porosa

XML Treatment for Cinachyrella paterifera

## Supplementary materials

Supplementary material 1 Table S1. Full collection details of each sample Data type: species data This is an open access article distributed under the terms of the Creative Commons Attribution License (CC BY 4.0), which permits unrestricted use, distribution, and reproduction in any medium, provided the original author and source are credited.Nadiezhda Santodomingo, Leontine E. Becking

Supplementary material 2 Figure S1. Type material of *Tethyamerguiensis* Data type: multimedia Explanation note: NHMUK 1894.11.16.17, Mergui Archipelago, Myanmar. **A** two slide preparations of the type specimen **B** skeleton, showing anatriaenes and oxeas **C** oxea, anatriaene, and protriaene **D** thin microxeas and sigmaspires **E** sigmaspires. Scale bars: 100 μm (**B, D**); 50 μm (**C**); 20 μm (**E**). This is an open access article distributed under the terms of the Creative Commons Attribution License (CC BY 4.0), which permits unrestricted use, distribution, and reproduction in any medium, provided the original author and source are credited.Nadiezhda Santodomingo, Leontine E. Becking
